# Multi‐Species Impacts of Invasive *Opuntia* Cacti on Mammal Habitat Use

**DOI:** 10.1111/ele.70163

**Published:** 2025-07-02

**Authors:** Peter S. Stewart, Russell A. Hill, Ayub M. O. Oduor, Philip A. Stephens, Mark J. Whittingham, Wayne Dawson

**Affiliations:** ^1^ School of Mathematics and Statistics University of Glasgow Glasgow UK; ^2^ Department of Biosciences Durham University Durham UK; ^3^ Department of Anthropology Durham University Durham UK; ^4^ SARChI Chair on Biodiversity Value and Change in the Vhembe Biosphere Reserve, Faculty of Science, Engineering and Agriculture University of Venda Thohoyandou South Africa; ^5^ Department of Biological and Life Sciences Technical University of Kenya Nairobi Kenya; ^6^ School of Natural and Environmental Sciences Newcastle University Newcastle upon Tyne UK; ^7^ Department of Evolution, Ecology and Behaviour, Institute of Infection, Veterinary and Ecological Sciences University of Liverpool Liverpool UK

**Keywords:** activity, animal behaviour, behavioural effects, biological invasions, camera traps, causal inference, invasive plants, occupancy

## Abstract

Biological invasions impact ecosystems worldwide, including through changing the behaviour of native species. Here, we used camera traps to investigate the effects of invasive *Opuntia* spp. on the habitat use of 12 mammal species in Laikipia County, Kenya, an internationally important region of mammalian biodiversity. We found that *Opuntia* impacted mammal occupancy and activity. These effects were evident when *Opuntia* was considered at both site level and landscape scales; however, some mammal species showed different responses to increasing *Opuntia* at these two scales. The effects of *Opuntia* were generally context dependent, with their strength and direction varying among mammal species and between seasons. As well as having important implications for mammal conservation, ecosystem functioning and the future spread of *Opuntia*, our findings highlight behavioural changes in large mammals as a potentially important pathway through which invasive species impact ecosystems.

## Graphical abstract

Biological invasions impact ecosystems worldwide, including through changing the behaviour of native species. Here, we used camera traps to investigate the effects of invasive *Opuntia* spp. on the habitat use of 12 mammal species in an internationally important region of mammalian biodiversity. We found clear effects of *Opuntia* on the occupancy and activity of multiple species, with the strength and direction of effects varying according to spatial scale and environmental context.

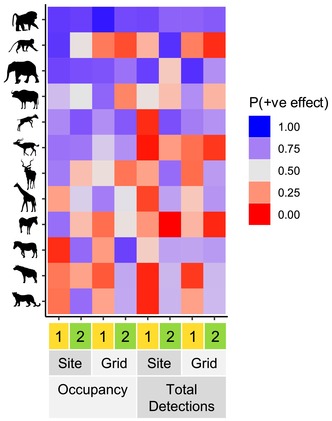

## 1 Introduction

Biological invasions are a rapidly expanding threat to ecosystems worldwide (Roy et al. 2024). Understanding the impacts of invasive species is vital if we are to mitigate them effectively but, until recently, attention has focused on impacts on biodiversity and native species' abundance (Crystal‐Ornelas and Lockwood 2020). However, a growing body of evidence now indicates that invasive species can cause profound ecological impacts by altering the behaviour of native animals (Langkilde et al. 2017; Stewart et al. 2021). Research to date indicates that these behavioural impacts can be highly scale and context‐dependent; improving our understanding of how impacts vary across spatial scales, and which aspects of environmental context predict the direction and magnitude of behavioural change, are key research questions in the field (Stewart et al. 2021).

Large mammals can be an important component of terrestrial ecosystems (Pringle et al. 2023; Ripple et al. 2014). Large mammalian herbivores structure the composition and trait distributions of plant communities (Boulanger et al. 2018; Dantas and Pausas 2020; Jia et al. 2018), disperse nutrients (le Roux et al. 2020) and seeds (Campos‐Arceiz and Blake 2011), and regulate fire regimes (Karp et al. 2024), while large mammalian carnivores play a key role in controlling herbivore and mesopredator populations, with indirect effects on a range of ecosystem processes (Ripple et al. 2014; Ritchie and Johnson 2009). Importantly, behaviour can moderate the ecological effects of large mammals (Pringle et al. 2023). Consequently, changes to large mammal behaviour caused by invasive species may constitute an important, yet underappreciated, impact pathway for biological invasions.

Laikipia County, Kenya, is a key stronghold for mammalian biodiversity and hosts vital populations of endangered mammals including Grevy's zebra ( *Equus grevyi* ; Rubenstein et al. 2016) and reticulated giraffe ( *Giraffa reticulata* ; Muneza et al. 2018). However, the region is also undergoing invasion by prickly pear cacti (*Opuntia* spp.). Native to the Americas, several species of *Opuntia* were introduced to Laikipia County, Kenya, in the latter half of the 20th century to serve as live fences and ornamental plants (Loisaba Conservancy 2019; Strum et al. 2015; Witt 2017). Since their introduction, three of these species ( *O. stricta* , *O. engelmannii* and *O. ficus‐indica* ) have become invasive, spreading rapidly to cover large areas of the landscape (Githae 2019; Strum et al. 2015; Witt 2017). Although biological control using cochineal ( *Dactylopius opuntiae* ) has been employed with some success to counteract the spread of *O. stricta* (Shackleton et al. 2017; Witt et al. 2020), the effectiveness against other *Opuntia* species—particularly *O. engelmannii* —appears to be limited. In addition to presenting an important applied conservation problem—with global relevance due to the worldwide invasion success of *Opuntia* species (Foxcroft et al. 2004; Pasiecznik and Rojas‐Sandoval 2007; Pasiecznik 2015)–the ongoing spread of *Opuntia* in Laikipia County provides a natural experiment to assess the impact of plant invasions on the behaviour of a suite of wild mammals.

*Opuntia* invasions could alter mammal behaviour through two mutually non‐exclusive pathways. First, *Opuntia* substantially alters the physical structure of the habitat by forming dense, impenetrable stands (Witt 2017). These stands may disrupt sightlines and restrict movement, altering patterns of actual or perceived predation risk. *Opuntia* stands may also impede herbivores' access to forage (Oduor et al. 2018); this effect is likely to be especially pronounced in drought conditions, when the overall availability of forage decreases (Boutton et al. 1988). Second, mature *Opuntia* stands provide a year‐round supply of fruit which is consumed by species including elephants ( *Loxodonta africana* ), olive baboons, ( *Papio anubis* ) and vervet monkeys ( *Chlorocebus pygerythrus* ; Githae 2019; Strum et al. 2015; Witt 2017). Consequently, these species may be attracted to invaded areas (Shackleton et al. 2017), which could increase seed dispersal and accelerate the invasion.

The ongoing spread of a globally significant plant invader in a region where large mammals are both diverse and ecologically important (Pringle et al. 2010) provides an opportunity to make use of a ‘natural experiment’ (the varying impacts across a landscape of *Opuntia* on vegetation structure and food abundance) to deepen our fundamental understanding of the behavioural impacts of invasive species. Here, using camera traps and drawing on insights from the field of causal inference, we quantified the total effects of *Opuntia* on the occupancy, total activity and temporal activity patterns of 12 mammal species which represent a range of trophic guilds and body sizes. To improve our understanding of the roles of spatial scale and environmental context in governing the behavioural impacts of plant invasions (Stewart et al. 2021), we examined species' responses to both the site‐level *Opuntia* cover and the quantity of *Opuntia* in the surrounding landscape, and explored how the effects varied across two seasons. Finally, to gain further insights into the mechanisms underlying the behavioural impacts of plant invasions, we examined whether *Opuntia* exerts indirect effects on habitat use through changing the cover of native plant species.

## 2 Methods

### 2.1 Study System

**FIGURE 1:**
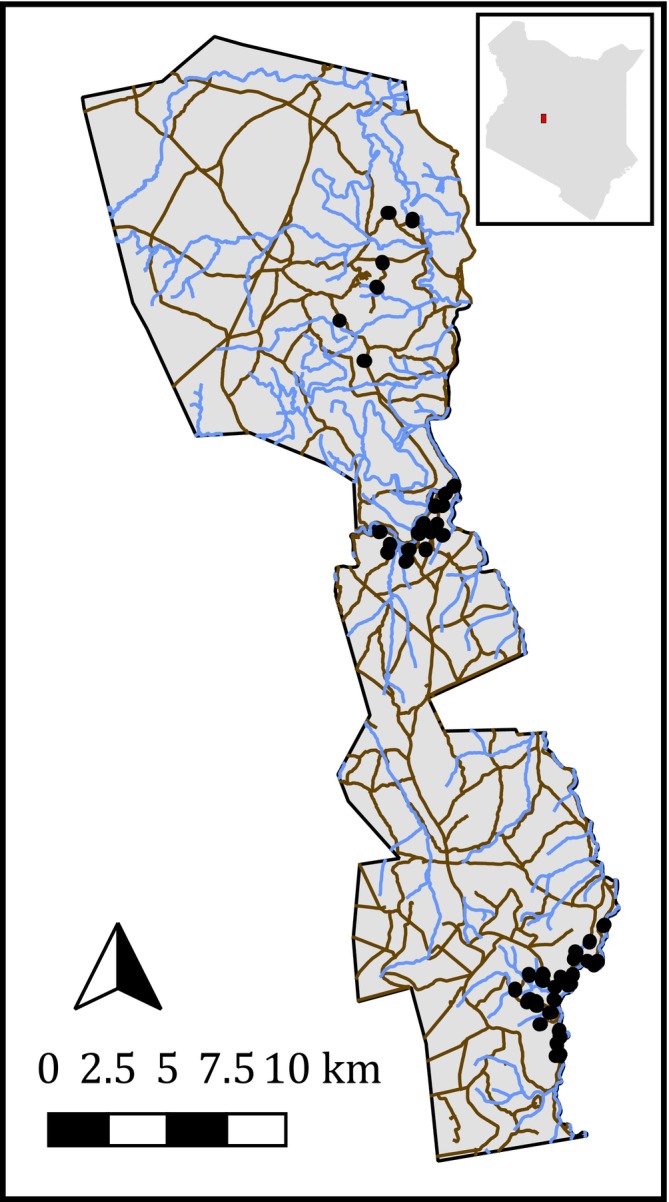
Map of the study area. Black points represent camera trap sites, light blue lines are rivers and brown lines are roads. Camera trap sites are clustered within three regions: Loisaba (top), Mpala north (middle) and Mpala south (bottom). Canvas extends from easting 244165 to 271026 m, northing 28814 to 78171 m (CRS: EPSG: 21097). Inset shows location of the study region within Kenya.

We conducted our study at Mpala Research Centre and Loisaba Conservancy, in Laikipia County, Kenya (Figure 1, see Supporting Information for detailed description). We focused on 12 mammal species. Olive baboons ( *P. anubis* ), vervet monkeys ( *C. pygerythrus* ) and elephants ( *L. africana* ) were selected because they commonly feed on *Opuntia* fruit and are presumed to be important dispersal agents (Githae 2019; Strum et al. 2015; Witt 2017). Buffalo ( *Syncerus caffer* ), dik‐dik (*Madoqua* spp.), impala ( *A. melampus* ), greater kudu ( *Tragelaphus strepsiceros* ), reticulated giraffe ( *G. reticulata* ), Grevy's zebra ( *E. grevyi* ) and plains zebra ( *E. quagga* ) were selected to represent a range of body sizes and diet compositions (Kartzinel et al. 2015; Kartzinel et al. 2019) across herbivore species which are not known to feed on *Opuntia* fruit. Finally, we included two carnivore species: spotted hyena ( *Crocuta crocuta* ) and leopard ( *Panthera pardus* ).

### 2.2 Camera Trap Deployment

To explore the effects of *Opuntia* on mammals' occupancy and activity, we deployed camera traps (see Supporting Information) in three sub‐regions of the study area (Figure 1) from January–April to October–November 2021. We selected the sub‐regions because each contained varying densities of *Opuntia*, from scattered individual plants to heavily invaded areas where *Opuntia* covered the majority of the ground (Figure S1). To maximise variation in *Opuntia* while minimising variation in confounding variables, we employed a paired‐site design, where a site is defined as the area immediately adjacent to one camera trap. Each sub‐region was divided into 500 × 500 m grid squares, with a subset of squares randomly selected for sampling. We placed two cameras within each square; the first camera was deployed in an area visually identified as high *Opuntia* density, and the second was deployed in a random direction 50–70 m away. If the second site was found to have an equal or higher *Opuntia* density than the first site, we generated a new random direction until the density at the second site was lower. In total, we sampled 101 sites within 46 squares for January–April, and 27 sites within 14 squares for October–November.

### 2.3 Habitat Surveys and Estimation of Grid Square‐Level Opuntia

To collect information on site‐level variables that could affect occupancy and activity, we conducted habitat surveys (see Supporting Information) in a circular area with 10 m radius, centred on the camera. We estimated the percentage of ground covered by *Opuntia* spp., grasses, shrubs, forbs, succulents, trees, bare ground and other cover (e.g., rocks) using a cover estimator chart (Anderson 1986). These percentages were not required to sum to 100%, as vegetation types could grow under/over one another. In addition, we counted the number of standing trees (woody plants taller than 2 m; shorter woody plants were classed as shrubs). To quantify the use of each site by livestock, we calculated the proportion of days in which livestock were detected by the camera. Finally, we calculated the straight‐line distances from each site to the nearest river and road using QGIS (v2.28.25; QGIS Development Team 2018).

To estimate the quantity of *Opuntia* spp. in each grid square, we performed distance sampling (Kéry and Royle 2015; see Supporting Information). Due to constraints imposed by the COVID‐19 pandemic, we only sampled 41 of the 46 squares in which camera traps were deployed. We first used a Poisson‐binomial multinomial distance sampling model with half‐normal detection function (Kéry and Royle 2015; code adapted from Joseph 2021) to estimate abundance for three *Opuntia* size classes (small = < 1 m, medium = 1–2 m, large = > 2 m height). We then combined the abundance estimates for different size classes into a single volume estimate by assuming (based on the volume of a hemisphere) that the volume of a large stand (h = 2.5 m) was 32.725 m^3^, a medium stand (h = 1.5 m) was 7.070m^3^ and a small stand (h = 0.5 m) was 0.260 m^3^. Finally, we divided each square's volume estimate by the respective transect length (measured using QGIS) to obtain the volume per metre of transect. Where estimates of the total grid square *Opuntia* volume were required, we multiplied the volume per metre of transect by 500.

### 2.4 Processing Camera Trap Images

We used Megadetector (v.4.1.0, Beery et al. 2019) to classify images as containing an animal (any species), human or vehicle. We manually screened all images with probability of ≥ 0.10 of containing a human or vehicle, discarding all images which contained a human or vehicle and retaining images which contained animals. We also retained all images classed as containing at least one animal with probability ≥ 0.98.

We uploaded all retained images to the Zooniverse platform (Prickly Pear Project Kenya: https://www.zooniverse.org/projects/peter‐dot‐stewart/prickly‐pear‐project‐kenya), where members of the public were able to classify the images. Each image was classified by at least 12 volunteers before retirement from the active image pool, except for images classified as ‘human’, which were immediately retired. We generated consensus classifications for each image using a threshold‐based approach, in which at least 66% of the volunteers had to classify the species as being present. We also quantified volunteer agreement by calculating the Shannon entropy (Shannon 1948) of the classification distribution for each image; images with entropy values greater than one were discarded. In cases where an image was classified by an expert (either P.S.S. or the Prickly Pear Project Kenya moderator), the expert classification was accepted regardless of volunteer disagreement.

### 2.5 Statistical Models

We fitted models to explore the effects of *Opuntia* on three aspects of habitat use. First, we used an occupancy model (MacKenzie et al. 2002) to explore the effects on occupancy (i.e., the probability that a site is used at least once). Second, we modelled the total number of daily detections using a negative‐binomial model. Finally, we used a binomial model to explore temporal shifts in species' activity by modelling the proportion of detections occurring at night (after dusk and before dawn; times obtained using the *suncalc* package; v.0.5.1; Thieurmel and Elmarhraoui 2022).

**FIGURE 2:**
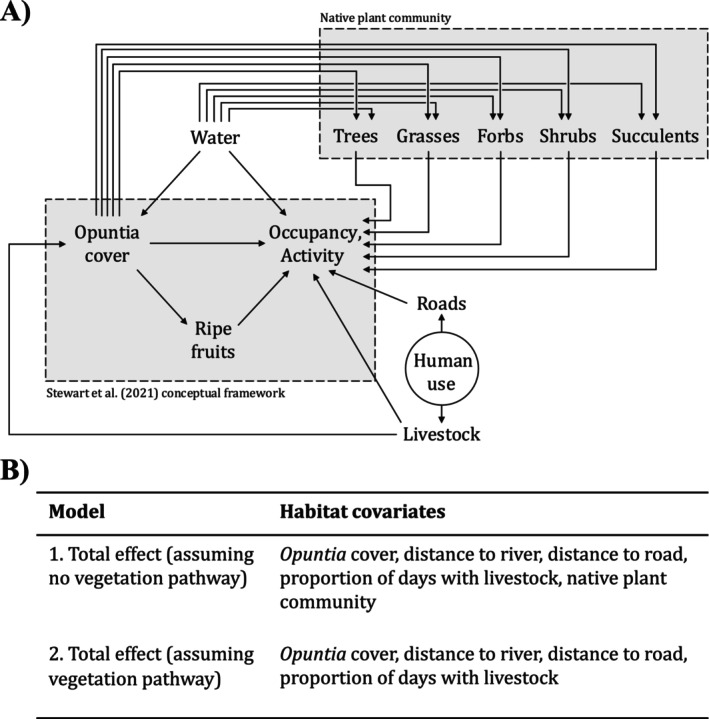
(A) Directed acyclic graph representing assumptions about the ways in which *Opuntia* cover and other environmental variables might affect mammal occupancy and activity. Nodes represent variables, while arrows represent possible mechanistic links between variables. ‘Human use’ is a latent variable and is therefore displayed in a circle. (B) Habitat covariates included in our occupancy, negative‐binomial, and binomial models. ‘Native plant community’ is shorthand for the total percentage cover covariates for grass, shrubs, forbs and succulents, and the total number of trees.

We estimated the total effect (i.e., including both the direct and indirect pathways; Figure 2A) of *Opuntia* measured at two spatial scales: the site‐level *Opuntia* cover and the grid square‐level *Opuntia* volume. In both cases, we estimated separate effects for the January–April and October–November seasons. We selected additional covariates by encoding our causal assumptions about the data‐generating process in a directed acyclic graph (Figure 2A) and selecting variables to satisfy the back‐door criterion (Arif and MacNeil 2022; Pearl 1995; Stewart, Stephens, et al. 2023). As with the *Opuntia* effect, we estimated separate covariate effects for each season. Furthermore, we fitted two sets of models (Figure 2B) to examine whether *Opuntia* indirectly affects occupancy and activity through its effects on the native plant community.

In addition to *Opuntia* and the habitat covariates (Figure 2B), in our occupancy model we included daily mean temperature in the detection sub‐model, as changes to the thermal environment can affect the performance of the camera's passive infrared sensor (Welbourne et al. 2016). We also included an offset intercept for each model of camera trap. In our binomial model, we included an interaction between *Opuntia* and lunar illumination (obtained using the *suncalc* package), to investigate whether moonlight moderated *Opuntia*'s effects (e.g., by altering predation risk; Prugh and Golden 2014). We standardised all covariates, excluding lunar illumination, by subtracting the mean and dividing by the standard deviation. In all models, we incorporated a Gaussian process to model spatial autocorrelation.

We fitted all models in a Bayesian framework using Stan, implemented with CmdStan (v2.33.1; Stan Development Team 2023); all data processing and visualisation was performed in R (v.4.4.2; R Core Team 2024). We used weakly regularising prior distributions (McElreath 2021, 214). For each model we used four chains, each with 2000 sampling iterations. We ran 4000 warmup iterations for the occupancy and binomial models, and 7000 for the negative‐binomial model. We assessed convergence using the $\hat{R}$ diagnostic (Vehtari et al. 2021), inspected the effective sample size for each parameter, and checked for divergent transitions.

## 3 Results

We collected a total of 1,726,954 camera trap images. After retaining images which Megadetector classified as containing at least one animal, and discarding images which contained humans or vehicles, the remaining 186,861 images were uploaded to Zooniverse. We were able to obtain at least one consensus classification for 174,336 of these images; of these, 22,714 images were consensus classified as empty (i.e., Megadetector false positives). For 12,525 of the images uploaded to Zooniverse, we were unable to obtain a consensus classification, generally because the animal was too poorly photographed to be identified to species level. As some images contained multiple species, we ultimately obtained 155,630 species detections across 151,622 images. Based on the analysis of 26,952 images which were classified by an expert in addition to the volunteers, our approach resulted in accurate classifications, with sensitivity ≥ 0.973 and specificity ≥ 0.998 for all focal species (Table S1).

**FIGURE 3:**
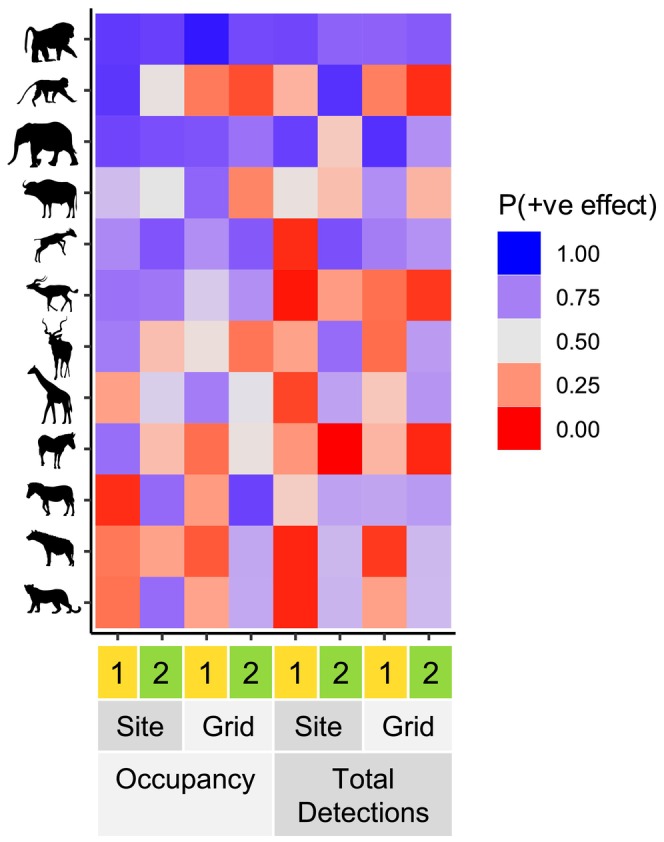
Proportion of posterior probability lying above zero (P(+ve effect)) for the effect of *Opuntia* on 12 mammal species. As this proportion increases towards 1.00 (blue cells) the probability of a positive effect is higher, while as the proportion decreases towards 0.00 (red cells) a negative effect is more probable. A value of 0.50 indicates that positive and negative effects are equally probable. Rows indicate species (from top: olive baboon, vervet monkey, elephant, buffalo, dik‐dik, impala, kudu, giraffe, Grevy's zebra, plains zebra, spotted hyena, leopard). Columns indicate combinations of season (1 = January–April, 2 = October–November), spatial scale of *Opuntia* covariate (site‐level % cover, grid square‐level volume), and model type (occupancy, total number of detections per day). Only one species (Olive Baboon) showed consistent (positive) effects across all seasons, sites and measures of occupancy/abundance. All other species showed variation in the direction of the effects of *Opuntia* to some degree.

We found that the effects of *Opuntia* on occupancy and the total number of daily detections were evident for multiple species. However, the strength and direction of these effects varied among species, between seasons, and according to the spatial scale of *Opuntia* covariate (Figure 3). In general, the effects were similar regardless of whether measures of the native plant community were included in the model (Figures S2 and S3). We also found that in addition to the effects on occupancy and total detections, increasing *Opuntia* was associated with a shift in the proportion of detections occurring at night for some species, with these effects being moderated by season and lunar illumination in several cases (see Supplementary Results, Figures S4–S7).

### 3.1 Occupancy

**FIGURE 4:**
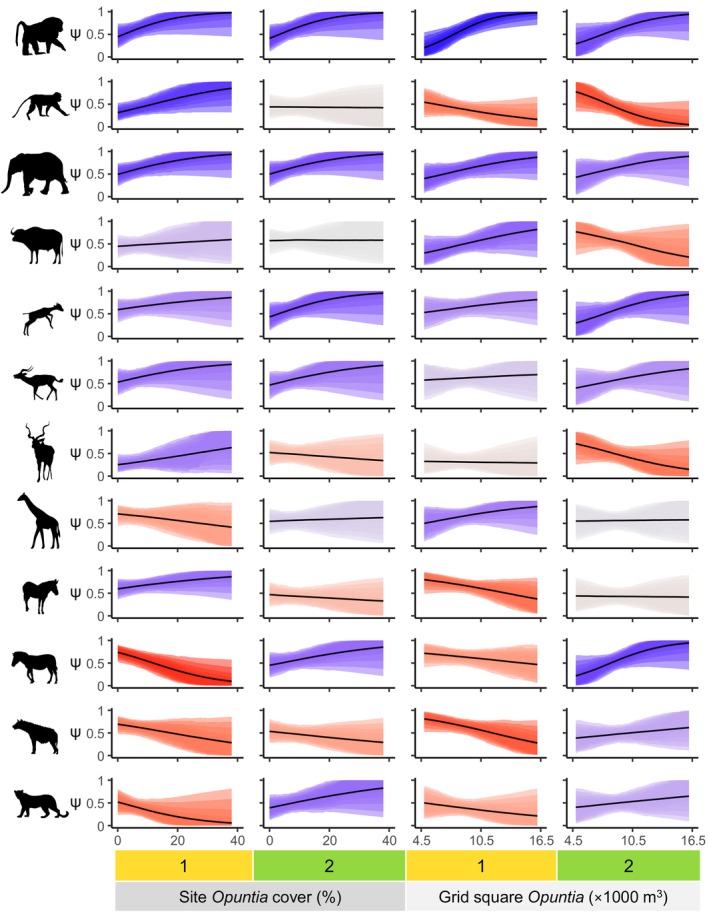
Marginal effects of *Opuntia* on occupancy probability (ψ) for 12 mammal species (from top: olive baboon, vervet monkey, elephant, buffalo, dik‐dik, impala, kudu, giraffe, Grevy's zebra, plains zebra, spotted hyena, leopard). Columns indicate combinations of season (1 = January–April, 2 = October–November) and spatial scale of *Opuntia* covariate (site‐level % cover, grid square‐level volume). Shaded regions represent (from outside) 95%, 89%, 80%, 70%, 60% and 50% compatibility intervals, with colours representing the proportion of posterior probability lying above zero for the *Opuntia* effect (see Figure 3 legend).

In our occupancy models for olive baboons and elephants, we observed positive effects of *Opuntia* for both seasons and spatial scales (Figure 4). By contrast, the effect for vervet monkeys was only positive for site‐level *Opuntia* in January–April; in October–November, we observed no clear effect, and for grid square *Opuntia* the estimated effect was negative in both seasons (Figure 4).

For the non‐frugivorous herbivores, we observed a variety of effects of *Opuntia* on occupancy. While buffalo occupancy did not exhibit a clear relationship with site‐level *Opuntia* in either season, their occupancy was positively related to grid square *Opuntia* in January–April and negatively related in October–November (Figure 4). For both dik‐dik and impala, the effect of *Opuntia* was positive at both spatial scales, but with generally stronger evidence for a positive effect in October–November (Figure 4). By contrast, the estimated effects for kudu were positive for site‐level *Opuntia* in January–April and negative for grid square *Opuntia* in October–November; we did not observe clear effects for kudu in the other two cases (Figure 4). For reticulated giraffe, the effect of site‐level *Opuntia* was negative in January–April, and there was a positive effect of grid square *Opuntia* in the same season (Figure 4). Grevy's zebra occupancy exhibited a positive relationship with site‐level *Opuntia* and a negative relationship with grid square *Opuntia* in January–April; we did not observe any clear effects for October–November (Figure 4). By contrast, the effect of *Opuntia* on plains zebra occupancy was negative at both spatial scales in January–April but positive at both scales in October–November (Figure 4).

Spotted hyena and leopard occupancies were negatively related to *Opuntia* at both spatial scales in January–April (Figure 4). However, the effects in October–November were positive (albeit uncertain) for both species in the grid square model, and for leopard in the fine‐scale model. The effect for hyena in the site‐level model was weakly negative.

### 3.2 Total Number of Detections

**FIGURE 5:**
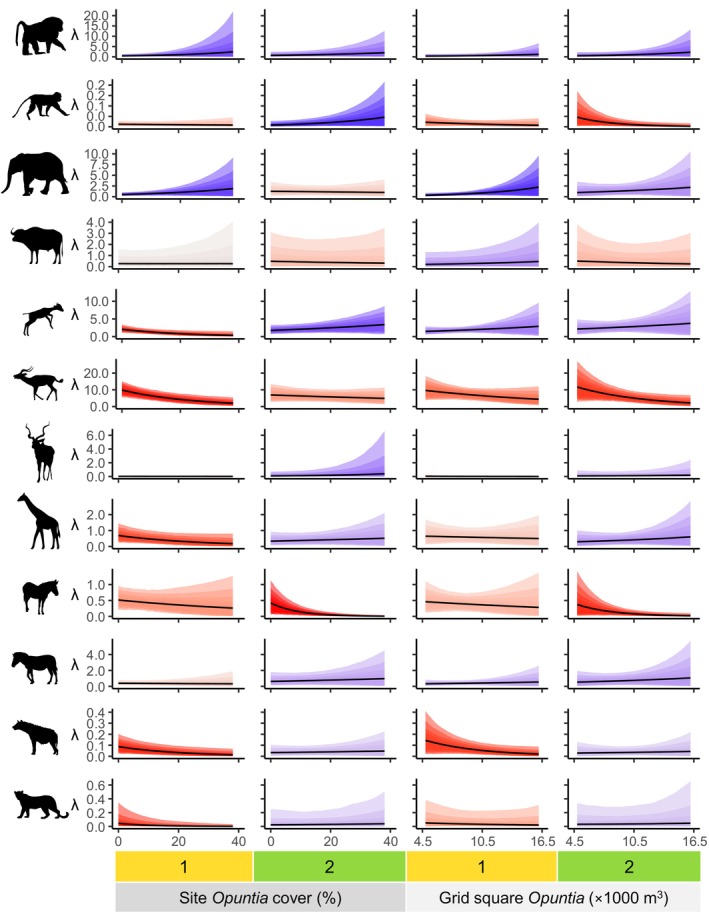
Marginal effects of *Opuntia* on expected total number of detections per day (λ) for 12 mammal species (from top: olive baboon, vervet monkey, elephant, buffalo, dik‐dik, impala, kudu, giraffe, Grevy's zebra, plains zebra, spotted hyena, leopard). Columns indicate combinations of season (1 = January–April, 2 = October–November) and spatial scale of *Opuntia* covariate (site‐level % cover, grid square‐level volume). Shaded regions represent (from outside) 95%, 89%, 80%, 70%, 60% and 50% compatibility intervals, with colours representing the proportion of posterior probability lying above zero for the *Opuntia* effect (see Figure 3 legend).

In our models for the total number of daily detections, the estimated effects of *Opuntia* were small and uncertain in many cases (Figure 5). Nevertheless, we did observe clear effects for some species.

Olive baboon and elephant detections increased with *Opuntia* at both scales (Figure 5), except for elephants and site‐level *Opuntia* in October–November. By contrast, in October–November vervet monkey detections were negatively related to grid square *Opuntia*, but positively related to site‐level *Opuntia*; the effects for January–April were very weak but probably negative (Figure 5).

Dik‐dik and impala exhibited clear negative effects of site‐level *Opuntia* in January–April, but not October–November (Figure 5). Notably, the effect of site‐level *Opuntia* on dik‐dik in January–April changed when measures of the native plant community were omitted (Figure S3), switching from negative to positive. For dik‐dik, the posterior median effect in the latter season was positive, while for impala the effect was weakly negative but quite uncertain. Grid square *Opuntia* volume had a clear negative effect on impala detections in October–November. The other effects of grid square *Opuntia* on dik‐dik and impala were less certain, but the posterior median effects were positive for dik‐dik and negative for impala. Reticulated giraffe detections showed a weak negative relationship with site‐level *Opuntia* in January–April (Figure 5). Grevy's zebra detections were also negatively related to *Opuntia* at both scales, but the effects were only clear in October–November (Figure 5). Finally, we observed clear negative effects at both scales for spotted hyena in January–April, and for leopards at a site level in the same season (Figure 5).

## 4 Discussion

We assessed the impacts of invasive *Opuntia* cacti on the habitat use of 12 native mammal species in Laikipia County, Kenya, which is a key stronghold for mammalian biodiversity. We found clear effects of *Opuntia* on the occupancy and activity of multiple species. These effects were evident when *Opuntia* was considered at both the site‐level and landscape scales. However, the magnitude and direction of effects differed according to species and season, while for some mammal species, effects differed between scales. Across all species, our results were similar regardless of whether we included measures of the native plant community in our models, suggesting that *Opuntia*'s effects are not mediated by changes to native vegetation cover. Our findings have implications for native plant community composition and functioning, the future spread of the *Opuntia* invasion, the conservation of endangered mammal species and human‐wildlife interactions.

### 4.1 Impacts of *Opuntia* Depend on Spatial Scale

The behavioural impacts of invasive plants can be highly scale‐dependent, with the direction and magnitude of effects potentially differing depending on whether they are considered at a local or landscape scale (Stewart et al. 2021). Consequently, we measured the effects of *Opuntia* on occupancy and activity at both a local (site‐level) and landscape (500 × 500 m grid square) scale. We found that the effects of *Opuntia* on mammal occupancy and activity were evident at both scales. However, the magnitude and, in some cases, the direction of effects varied between scales. For example, vervet monkey occupancy exhibited a positive or neutral relationship (depending on season) with site‐level *Opuntia* cover, but a consistently negative relationship with grid square‐level *Opuntia* volume. By contrast, olive baboon occupancy displayed a strong positive relationship with *Opuntia* at both spatial scales. The scale‐dependent effect for vervet monkeys may be attributable to competition with and the risk of predation from baboons (Willems and Hill 2009), which suggests that scale‐dependency in impacts on occupancy could arise from other native species' responses to plant invasion. More generally, the scale‐dependency of the effects for some of the species in our study underscores the importance of incorporating spatial scale into assessments and predictions of invasive species' ecological impacts (Kumschick et al. 2015).

### 4.2 Opuntia Impacts Depend on Species and Seasonal Context

The behavioural impacts of plant invasions are often highly context‐dependent; improving our understanding of this context dependency, including how behavioural effects can be predicted using information on species' traits and known axes of environmental variation, is a key fundamental research problem (Stewart et al. 2021). In our study, we found that *Opuntia*'s effects on occupancy and activity varied among native species. Importantly, some of the main differences in species' responses are consistent with information on the species' traits and natural histories, which supports the view that this information can be used to predict the impacts of recently introduced or emerging invasive plants (Stewart et al. 2021). For instance, we found differences in the responses of two of the region's most abundant herbivores—dik‐dik and impala—to increasing *Opuntia*; dik‐dik occupancy and daily detections were positively related to *Opuntia*, while impala daily detections decreased in invaded areas. These findings align with the species' preferences for open versus closed habitats; dik‐dik prefer closed habitats because they perceive open areas as risky, while impala tend to avoid closed habitats as they are less able to detect and escape from predators (Epperly et al. 2021; Ford et al. 2014; Otieno et al. 2019). Although the positive effect of *Opuntia* on impala occupancy initially appears inconsistent with this explanation, the discrepancy could be explained by invaded areas being occupied by poor‐condition individuals (Mcnamara and Houston 1987; Winnie and Creel 2007) or bachelor herds. Additionally, dik‐dik sometimes consume *Opuntia* flowers (Stewart 2023), which may further attract dik‐dik to invaded areas.

In addition to the differences among native species, we also observed differences between seasons. In particular, we observed a seasonally‐varying effect of *Opuntia* on the occupancy of Grevy's zebra and plains zebra—two grazers which are not known to consume *Opuntia* fruit. In the predominantly dry January–April season, the occupancies of both species declined as the quantity of *Opuntia* in the grid square increased, while in the wetter October–November season the effect of *Opuntia* was neutral for Grevy's zebra and positive for plains zebra. These results may be explained by changes in forage availability; in wet conditions, when grass biomass is high (Boutton et al. 1988), there is ample forage for zebra to occupy heavily invaded areas. However, as grass biomass declines in dry conditions (Boutton et al. 1988), the areas between *Opuntia* stands no longer contain enough forage for zebra to persist, and they must instead concentrate on less invaded areas. This explanation is compatible with the fact that our models displayed similar results regardless of whether we included native vegetation (including grass) cover because grasses commonly grow among *Opuntia* stands, and hence contribute to percentage cover despite potentially being inaccessible to zebra. We also observed that the average number of Grevy's zebra detections declined with increasing *Opuntia* in both seasons; this may suggest that even though high‐*Opuntia* areas remain occupied, they are used less frequently. We did not observe similar effects for plains zebra; this difference could be explained by a number of factors, including differences in diet (Kartzinel et al. 2015; Kartzinel et al. 2019), water requirements (Tombak et al. 2022), and territoriality (Klingel 1975) between the two species.

### 4.3 No Evidence for an Indirect Effect of Opuntia Mediated by Native Vegetation Cover

When we compared models with and without native vegetation cover, we observed qualitatively similar results in almost all cases. Based on our assumptions about the relationships between *Opuntia*, occupancy and activity, and other variables in the system (Figure 2), this result suggests that *Opuntia* does not influence mammal occupancy and activity indirectly via altering the general composition of the native plant community (e.g., by inducing a transition from a grass‐dominated to shrub‐dominated plant community). Rather, we argue that *Opuntia* primarily affects occupancy and activity through changing the physical structure of the habitat, and by altering the distribution of resources (especially through fruit provision). An important caveat is that we only examined broad categories of native vegetation. Therefore, we cannot eliminate the possibility that *Opuntia* may exert indirect effects through altering composition within vegetation grasses (e.g., facilitating one species of grass over another). Further insights could be gained by investigating how *Opuntia* invasion alters the local plant community, and by examining the diet of animals inhabiting areas with different levels of *Opuntia*.

### 4.4 Ecological and Conservation Implications of Changes to Behaviour

The heterogeneous effects we observed across herbivore species suggest that *Opuntia* invasion can substantially alter the composition of the local herbivore community. Long‐term herbivore exclusion experiments—conducted in the same region as our study (Goheen et al. 2013; Young et al. 1997)—illustrate that these compositional changes could result in powerful indirect effects on a number of ecosystem properties. For instance, mesoherbivores—including impala, kudu, buffalo and zebra—strongly reduce total understory density (Goheen et al. 2013), while megaherbivores—especially elephants—regulate shrub and tree dynamics, reducing the cover of taller shrubs and influencing species composition (Augustine and Mcnaughton 2004; Goheen et al. 2013). Smaller herbivores also play a role in shrub and tree dynamics; dik‐dik suppress the growth rate and biomass of shrubs close to the ground and reduce the recruitment of shrubs into larger size classes (Augustine and Mcnaughton 2004). Exclusion experiments also illustrate that herbivores' effects extend beyond the plant community. For example, herbivores affect the nitrogen cycle, and these effects appear to differ between browsers and grazers (Coetsee et al. 2023). Finally, the herbivore exclusion experiments suggest that the effects of *Opuntia* on mammalian herbivores may feed back to influence the future dynamics of the *Opuntia* invasion; herbivore‐accessible plots have significantly fewer *Opuntia* plants than plots which exclude herbivores, which may indicate that herbivores inhibit *Opuntia* establishment via herbivory or trampling (Wells et al. 2023). Our study found a reduced activity of impala—one of the region's most abundant herbivores—in invaded areas which may act in concert with greater frugivore occupancy and subsequent *Opuntia* dispersal to shift vegetation further towards an *Opuntia*‐dominated state.

The effects of *Opuntia* are not only likely to be ecologically impactful in Laikipia County, but are also important from a conservation perspective. In particular, the reduction in Grevy's zebra occupancy and detections in high‐*Opuntia* areas under dry conditions is especially significant for conservation, because it implies that the ongoing *Opuntia* invasion may erode the zebras' ability to withstand the frequent–and often severe–droughts which the region experiences (Ndiritu 2021). As Laikipia County hosts approximately half of the world's Grevy's zebra population (Rubenstein et al. 2016), the synergistic effects of *Opuntia* invasion and drought may pose a threat to the species. Consequently, further investigation into the effects of *Opuntia* on Grevy's zebra presents a key avenue for future research.

Finally, the behavioural impacts of *Opuntia* have implications for human‐wildlife interactions in the region. For example, our finding of a positive relationship between *Opuntia* and elephant occupancy is consistent with local communities' observations that elephants are attracted to *Opuntia* fruit, resulting in elephants encroaching with increased frequency on grazing areas near *Opuntia* stands (Shackleton et al. 2017). In addition, we observed a shift towards nocturnal activity for elephants as site‐level *Opuntia* increased. This suggests that *Opuntia* invasion around agricultural areas could exacerbate crop foraging, which occurs at night (Graham et al. 2010).

### 4.5 Potential Limitations and Future Directions

A key assumption of our approach is that the relationship between *Opuntia* and mammal habitat use is due to *Opuntia* affecting habitat use, and not habitat use affecting the distribution of *Opuntia* (Figure 2). In the long‐term, this assumption may not be strictly true. For instance, long‐term herbivore exclosure plots have a higher density of *O. stricta* than control sites which are accessible to herbivores, implying that wild herbivores suppress *Opuntia* growth either directly by feeding on *Opuntia*, or indirectly by influencing *Opuntia*'s interactions with the native plant community (Wells et al. 2023). However, these effects likely operate on much longer timescales—months to years—than changes in animal behaviour, which can occur over hours and days. Therefore, we argue that the effects observed over the relatively short timescale of our study are best interpreted as *Opuntia*'s effects on mammalian habitat use. However, it is clear that deepening our understanding of the complex interplay between the wild mammal community and *Opuntia* is an important avenue for future research; this is especially true for *O. engelmannii* , which has remained relatively unstudied to‐date. These longer‐term studies could aim to establish whether feedback loops—which are a common feature of the behavioural impacts of invasive plants (Stewart et al. 2021)—are an important feature of the *Opuntia* invasion in Kenya.

## 5 Conclusions

We have shown that invasive *Opuntia* cacti are altering the occupancy and activity of mammals in a globally significant site of mammalian biodiversity. Both local (site‐level) and landscape scale *Opuntia* cover have evident effects, with some mammal species exhibiting different responses to *Opuntia* at these two scales. Additionally, the strength and direction of *Opuntia*'s effects varied among mammal species and between seasons. Our analyses suggest that the impacts of *Opuntia* are direct, rather than being mediated by changes to native vegetation cover. These results have important ecological and conservation implications for the region. More broadly, our findings underscore the role of animal behaviour—and the behaviour of large mammals specifically—in mediating the ecological impacts of biological invasions. As invasive species continue to spread worldwide, obtaining a stronger understanding of these behavioural impacts will become increasingly important.

## Author Contributions

Peter S. Stewart and Wayne Dawson designed the data collection protocol. Peter S. Stewart collected and analysed the data and wrote the first manuscript draft. All authors contributed substantially to revisions of the framework and manuscript.

## Peer Review

The peer review history for this article is available at https://www.webofscience.com/api/gateway/wos/peer‐review/10.1111/ele.70163.

## Supporting information


Data S1.

## Data Availability

The data and code that support the findings of this study are openly available on Zenodo at https://doi.org/10.5281/zenodo.14389489 and https://doi.org/10.5281/zenodo.14388983
