# Supplementary material for: Multi‐Species Impacts of Invasive Opuntia Cacti on Mammal Habitat Use
Source: Ecol Lett. 2025 Jul 2;28(7):e70163. doi: 10.1111/ele.70163 (PMC12218864; doi:10.1111/ele.70163)
Supplement: Supplementary file 1 — Data S1. [file ELE-28-0-s001.docx]

**Supplementary Material**

**Appendix S1 – Supplementary Methods**

***Study system***

The study area is predominantly unfenced (Crego *et al.* 2021) semi-arid savanna, with varying densities of woodland and shrubland dominated by *Vachellia* and *Senegalia* (formerly *Acacia*) species, including *V. etbaica, S. brevispica, S. mellifera, and V. gerrardii*, alongside other species including *Boscia augustifolia*, *Croton dichogamus,* and *Grewia* spp. (Augustine 2003; Augustine *et al.* 2011; Mutuku & Kenfack 2019; Young *et al.* 1995). Grasses belonging to the genera *Cynodon*, *Pennisetum*, *Digitaria* and *Sporobolus* are common in the understory, and a variety of forbs including *Plecranthus* spp., *Pollichia campestris*, *Portulaca* spp. and *Blepharis* spp. are also present (Young *et al.* 1995). *Euphorbia nyikae* and other succulents also occur in some areas (Augustine 2003).

The most common *Opuntia* species present in the study area are *O. stricta* and *O. engelmannii*; a few scattered *O. ficus-indica* individuals are also present, particularly on rocky outcrops in the central Mpala region. At the time of data collection, *O. stricta* was generally distributed at moderate densities in the southern Mpala region, with lower densities in the northern Mpala region and no individuals at Loisaba, while *O. engelmannii* was present at very high densities at Loisaba and moderate to low densities in the northern Mpala region, with no individuals present in the southern Mpala region. As we assumed that the modes of impact (Stewart *et al*. 2021) of different *Opuntia* species are the same (see Fig. 2 in main text), we aggregated *O. stricta* and *O. engelmannii* data when calculating *Opuntia* cover and volume (see main text).

***Camera trap deployment***

We deployed 30 camera traps (20 Browning Dark Ops Pro, 5 Browning Recon Force Extreme, and 5 Reconyx Hyperfire 2); three were lost to damage, leaving 27 operational at the end of the study. Due to these losses, and with some grid squares sampled more than once, our total sample comprised 101 sites within 46 squares for January-April, and 27 sites within 14 squares for October-November. The sites sampled in October-November were a subset of those sampled in January-April. Cameras remained operational at each location for between 3 and 56 days in January-April (Q1 = 18, median = 23, Q3 = 26 days) before being moved to a new location; we aimed to leave cameras in place for at least one week before they were moved, but on a few occasions (n = 5) cameras were moved earlier for logistical reasons. In October-November the cameras were not moved, and instead remained in place for between 33 and 46 days (Q1 = 36, median = 37, Q3 = 37 days).

We mounted the cameras on tree trunks/stumps (average ground-to-lens height = 81cm) and positioned them to ensure good visibility 10m in front of the camera. We set the cameras to take images with a five second delay between captures. For the Browning cameras we used the “long range” infrared flash setting, and used the default “optimised” infrared flash for the Reconyx cameras.

***Habitat surveys and estimation of grid square-level Opuntia***

To collect information on site-level variables that could affect occupancy and activity, we conducted habitat surveys in a circular area with 10m radius, centred on the camera. We divided this area into the field of view (FOV), defined as the area in which we could see the camera’s lens, and the rest of the area located beside and behind the camera. Within each zone we estimated the percentage of ground covered by *Opuntia* spp., grasses, shrubs, forbs, succulents, trees, bare ground, and other cover (*e.g.,* rocks) using a cover estimator chart (Anderson 1986). These percentages were not required to sum to 100%, as vegetation types could grow under/over one another. We then averaged the values for the FOV and non-visible area to obtain a site-level value for each ground cover type. In addition, we counted the number of standing trees (woody plants taller than 2m; shorter woody plants were classed as shrubs). To quantify the use of each site by livestock, we calculated the proportion of days in which livestock were detected by the camera. Finally, we calculated the straight-line distances from each site to the nearest river and road using QGIS (v2.28.25; QGIS Development Team, 2018).

To estimate the quantity of *Opuntia* in each grid square, we performed distance sampling (Kéry & Royle, 2015). Due to constraints imposed by the COVID-19 pandemic, we only sampled 41 of the 46 squares in which camera traps were deployed. The sampling was conducted on foot along a transect; we looked for *Opuntia* spp. visible from the transect in either direction. When an *Opuntia* stand was observed we recorded the size category (small = <1m, medium = 1-2m, large = >2m height) and measured the distance to the stand. Distances were measured using a tape measure for stands <10m away, and using a laser range finder (Leica Rangemaster CRF 2400-R, accurate to ±1m) for stands 10-80m from the transect. We did not count stands further than 80m away to avoid including stands situated outside the square.

To obtain *Opuntia* volume estimates for each of the 41 squares, we first used a Poisson-binomial multinomial distance sampling model with half-normal detection function (Kéry & Royle, 2015; code adapted from Joseph, 2021) to estimate abundance for each *Opuntia* size class. We then combined the median abundance estimates for different size classes into a single volume estimate by assuming (based on the volume of a hemisphere) that the volume of a large stand (h = 2.5m) was 32.725m^3^, a medium stand (h = 1.5m) was 7.070m^3^, and a small stand (h = 0.5m) was 0.260m^3^. Finally, we divided each square’s volume estimate by the respective transect length (measured using QGIS) to obtain the volume per metre of transect. Where estimates of the total grid square *Opuntia* volume were required, we multiplied the volume per metre of transect by 500.

***References***

Augustine, D.J. (2003). Spatial heterogeneity in the herbaceous layer of a semi-arid savanna ecosystem. *Plant Ecology*, 167, 319–331.

Augustine, D.J., Veblen, K.E., Goheen, J.R., Riginos, C. & Young, T.P. (2011). Pathways for Positive Cattle–Wildlife Interactions in Semiarid Rangelands. *Smithsonian Contributions to Zoology*, 55–71.

Crego, R., Wells, H., Ndung’u, K., Evans, L., Nduguta, R., Chege, M., *et al.* (2021). Moving through the mosaic: identifying critical linkage zones for large herbivores across a multiple‐use African landscape. *Landscape Ecology*, 36.

Joseph, M.B. (2021). *distance-sampling.stan.* Available at: https://gist.github.com/mbjoseph/960c3b259d007c81bebbaf9e5be1e250. Accessed 28/7/22.

Kéry, M., & Royle, J.A. (2015). *Applied hierarchical modeling in ecology: analysis of distribution, abundance and species richness in R and BUGS: volume 1: prelude and static models.* Academic Press, London, UK

Mutuku, P.M. & Kenfack, D. (2019). Effect of local topographic heterogeneity on tree species assembly in an Acacia-dominated African savanna. *Journal of Tropical Ecology*, 35, 46–56.

QGIS Development Team (2018). *QGIS Geographic Information System.* v.2.18.25.

Stewart, P.S., Hill, R.A., Stephens, P.A., Whittingham, M.J. & Dawson, W. (2021). Impacts of invasive plants on animal behaviour. *Ecology Letters*, 24, 891–907.

Young, T.P., Patridge, N. & Macrae, A. (1995). Long-Term Glades in Acacia Bushland and Their Edge Effects in Laikipia, Kenya. *Ecological Applications*, 5, 97–108.

**Appendix S2 – Supplementary Results**

***Diurnal and Nocturnal Detections***

The timing of elephant activity was strongly influenced by site-level *Opuntia*, with a higher proportion of nocturnal detections occurring at high-*Opuntia* sites in both seasons (Fig. S4C). We also observed positive relationships between elephant nocturnal detections and grid square *Opuntia* in October-November (Fig. S5C). During January-April the proportion of night-time buffalo detections was negatively related to site-level *Opuntia* when lunar illumination was high, but positively related to grid square *Opuntia* regardless of lunar illumination (Fig. S5D). Dik-dik were detected less often at night as both site-level and grid square level *Opuntia* increased in October-November, and these effects were stronger under a full moon (Figs. S4E, S5E). Impala were observed more often at night as site-level *Opuntia* increased during October-November, but only under high lunar illumination (Fig. S4F). Conversely, the proportion of night-time detections decreased with grid square *Opuntia*, again only when the moon was full. For kudu, we observed a positive effect of grid square *Opuntia* which was strongest under high lunar illumination (Fig S5G). For giraffe, the proportion of night-time detections increased with grid square *Opuntia* in January-April under high lunar illumination, and decreased in October-November (Fig S5H). For Grevy’s zebra, the effects of grid-level *Opuntia* were negative in January-April under high lunar illumination and in October-November under low lunar illumination, but positive in October-November when lunar illumination was high (Fig. S5I).

Again, we observed qualitatively similar results regardless of whether we included measures of the native plant community (Figs. S6, S7).

**Appendix S3 – Supplementary Figures and Tables**

**Table S1.** Classification accuracy for volunteer classifications, based on 26952 images which were classified by both an expert and volunteers. Results are shown for both consensus classifications (see main text) as well as the raw non-consensus volunteer classifications. Sensitivity and specificity are defined as Pr(classified present | truly present) and Pr(classified absent | truly absent) respectively. Species marked with an asterisk (*) were not present in the expert-classified set of images, and therefore do not have sensitivity values as they were never truly present.

|  | **Raw** | | **Consensus** | |
| --- | --- | --- | --- | --- |
|  | **Sensitivity** | **Specificity** | **Sensitivity** | **Specificity** |
| **Focal species** | |  |  |  |
| Buffalo *(Syncerus caffer)* | 0.785 | 0.999 | 1.000 | 1.000 |
| Dik-dik *(Madoqua spp.)* | 0.848 | 0.985 | 0.993 | 1.000 |
| Elephant *(Loxodonta africana)* | 0.948 | 0.999 | 0.995 | 1.000 |
| Greater kudu *(Tragelaphus strepsiceros)* | 0.638 | 0.999 | 1.000 | 1.000 |
| Grevy’s zebra *(Equus grevyi)* | 0.762 | 0.995 | 0.973 | 1.000 |
| Impala *(Aepyceros melampus)* | 0.834 | 0.991 | 0.996 | 0.999 |
| Leopard *(Panthera pardus)* | 0.650 | 1.000 | 1.000 | 1.000 |
| Olive baboon *(Papio anubis)* | 0.899 | 0.999 | 0.997 | 1.000 |
| Plains zebra *(Equus quagga)* | 0.913 | 0.991 | 0.990 | 0.998 |
| Reticulated giraffe *(Giraffa reticulata)* | 0.908 | 1.000 | 0.995 | 1.000 |
| Spotted hyena *(Crocuta crocuta)* | 0.737 | 1.000 | 0.991 | 1.000 |
| Vervet monkey *(Chlorocebus pygerythrus)* | 0.879 | 0.999 | 1.000 | 1.000 |
|  |  |  |  |  |
| **Other mammals** | |  |  |  |
| Aardvark *(Orycteropus afer)** | NA | 0.999 | NA | 1.000 |
| Aardwolf *(Proteles cristata)** | NA | 1.000 | NA | 1.000 |
| African wild dog *(Lycaon pictus)** | 0.636 | 1.000 | 1.000 | 1.000 |
| African wildcat *(Felis lybica)** | NA | 1.000 | NA | 1.000 |
| Bat | 0.500 | 1.000 | 0.941 | 1.000 |
| Black-backed jackal *(Canis mesomelas)* | 0.479 | 0.999 | 1.000 | 1.000 |
|  | 0.872 | 0.999 | 0.993 | 0.999 |
| Bushbuck *(Tragelaphus sylvaticus)* | NA | 1.000 | NA | 1.000 |
| Camel *(Camelus dromedarius)* | NA | 1.000 | NA | 1.000 |
| Caracal *(Caracal caracal)** | 0.083 | 1.000 | 0.500 | 1.000 |
| Cheetah *(Acinonyx jubatus)** | NA | 1.000 | NA | 1.000 |
| Civet *(Civettictis civetta)** | 0.083 | 0.994 | 0.500 | 1.000 |
| Dog (domestic)* | 0.402 | 0.999 | 0.962 | 1.000 |
| Duiker (tribe Cephalophini)* | 0.722 | 1.000 | 1.000 | 1.000 |
| Eland *(Taurotragus oryx)* | NA | 0.995 | NA | 1.000 |
| Genet *(Genetta spp.)* | 0.388 | 0.984 | 0.857 | 1.000 |
| Gerenuk *(Lotocranius walleri)** | 0.762 | 0.999 | 0.990 | 1.000 |
| Grant’s gazelle *(Nanger granti)* | NA | 0.999 | NA | 1.000 |
|  | 0.916 | 1.000 | 1.000 | 1.000 |
| Hare *(Lepus victoriae)* | 0.745 | 1.000 | 1.000 | 1.000 |
| Hartebeest *(Alcelaphus buselaphus)** | NA | 1.000 | NA | 1.000 |
| Hippopotamus *(Hippopotamus amphibius)* | 0.848 | 1.000 | 1.000 | 1.000 |
| Honey badger *(Mellivora capensis)* | 0.692 | 1.000 | 1.000 | 1.000 |
| Hyrax (family Procaviidae)* | 0.822 | 0.996 | 0.927 | 1.000 |
| Lion *(Panthera leo)* | 0.762 | 0.999 | 1.000 | 1.000 |
| Livestock (non-camel) | 0.575 | 1.000 | 1.000 | 1.000 |
| Mongoose (family Herpestidae) | 0.750 | 0.999 | 1.000 | 1.000 |
| Mouse/rat | 0.441 | 1.000 | 1.000 | 1.000 |
| Oryx *(Oryx beisa)* | 0.769 | 0.999 | 0.978 | 1.000 |
| Porcupine *(Hystrix cristata)* | NA | 0.997 | NA | 1.000 |
| Squirrel (tribe Xerini) | 0.563 | 1.000 | 0.933 | 1.000 |
| Steenbok *(Raphicerus campestris)** | NA | 0.998 | NA | 1.000 |
| Striped hyena *(Hyaena hyaena)* | 0.844 | 0.999 | 0.988 | 1.000 |
| Thompson’s gazelle *(Nanger granti)** | 0.548 | 0.999 | 0.989 | 1.000 |
| Warthog *(Phacochoerus spp.)* | 0.520 | 1.000 | 1.000 | 1.000 |
| Waterbuck *(Kobus defassa)* | NA | 0.999 | NA | 1.000 |
| Zorilla *(Ictonyx striatus)* | NA | 1.000 | NA | 1.000 |
|  |  |  |  |  |
| **Birds and other taxa** |  |  |  |  |
| Bird (other) | 0.806 | 0.995 | 0.975 | 0.999 |
| Helmeted guineafowl *(Numida meleagris)* | 0.738 | 0.996 | 0.977 | 1.000 |
| Insect/spider | 0.083 | 0.999 | 0.500 | 1.000 |
| Kori bustard *(Ardeotis kori)* | 0.783 | 0.999 | 1.000 | 1.000 |
| Ostrich *(Struthio camelus)** | NA | 1.000 | NA | 1.000 |
| Reptile/amphibian | 0.815 | 1.000 | 1.000 | 1.000 |
| Secretary bird *(Sagittarius serpentarius)* | 0.848 | 1.000 | 1.000 | 1.000 |
| Vulturine guineafowl *(Acryllium vulturinum)* | 0.901 | 0.995 | 0.997 | 1.000 |
|  |  |  |  |  |
| **Special categories** |  |  |  |  |
| Human | 0.214 | 1.000 | 1.000 | 1.000 |
| Other | 0.267 | 0.990 | 0.667 | 1.000 |
| No animals present | 0.954 | 0.969 | 0.994 | 0.996 |
|  |  |  |  |  |

**A)**


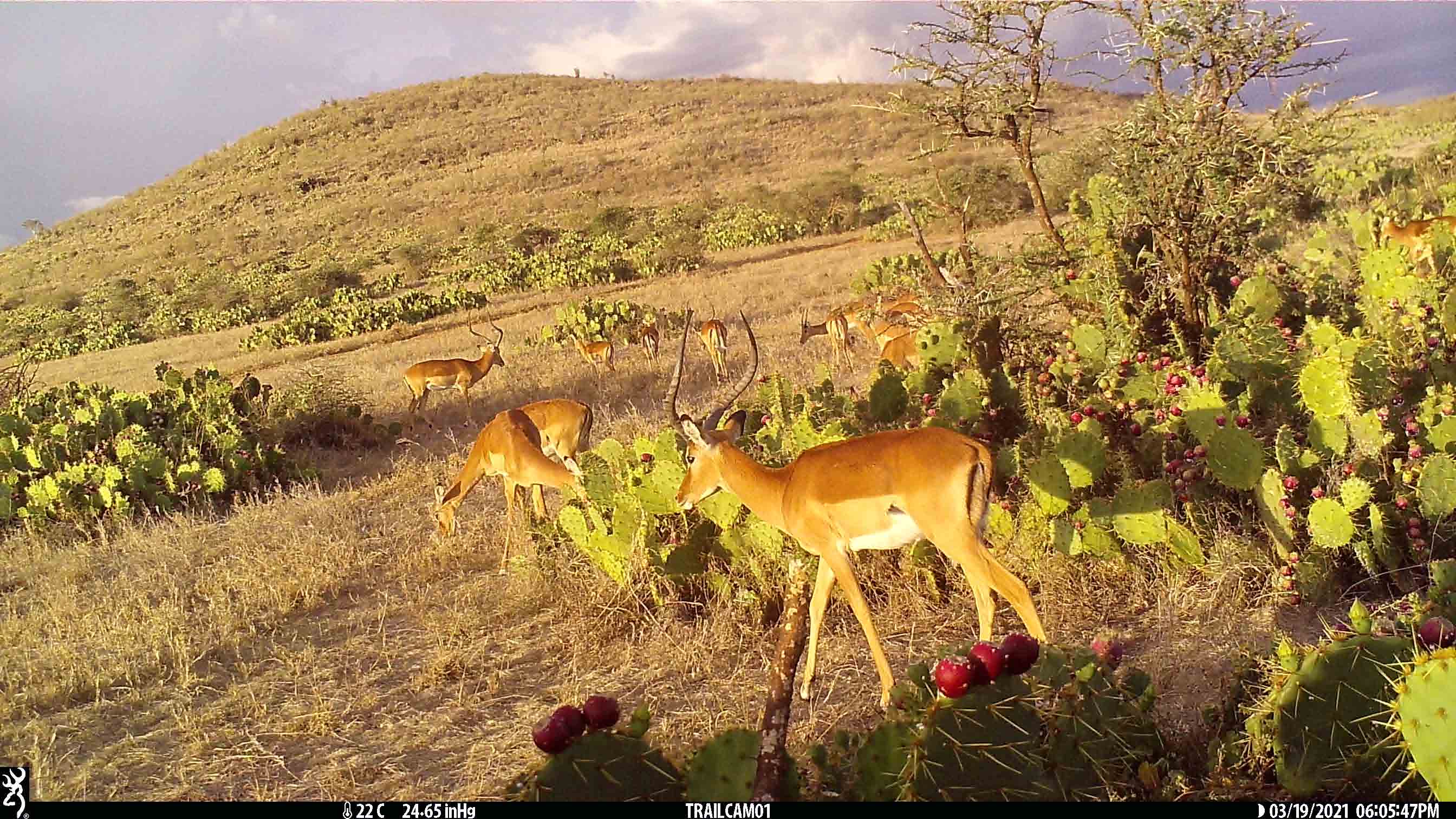


**B)**


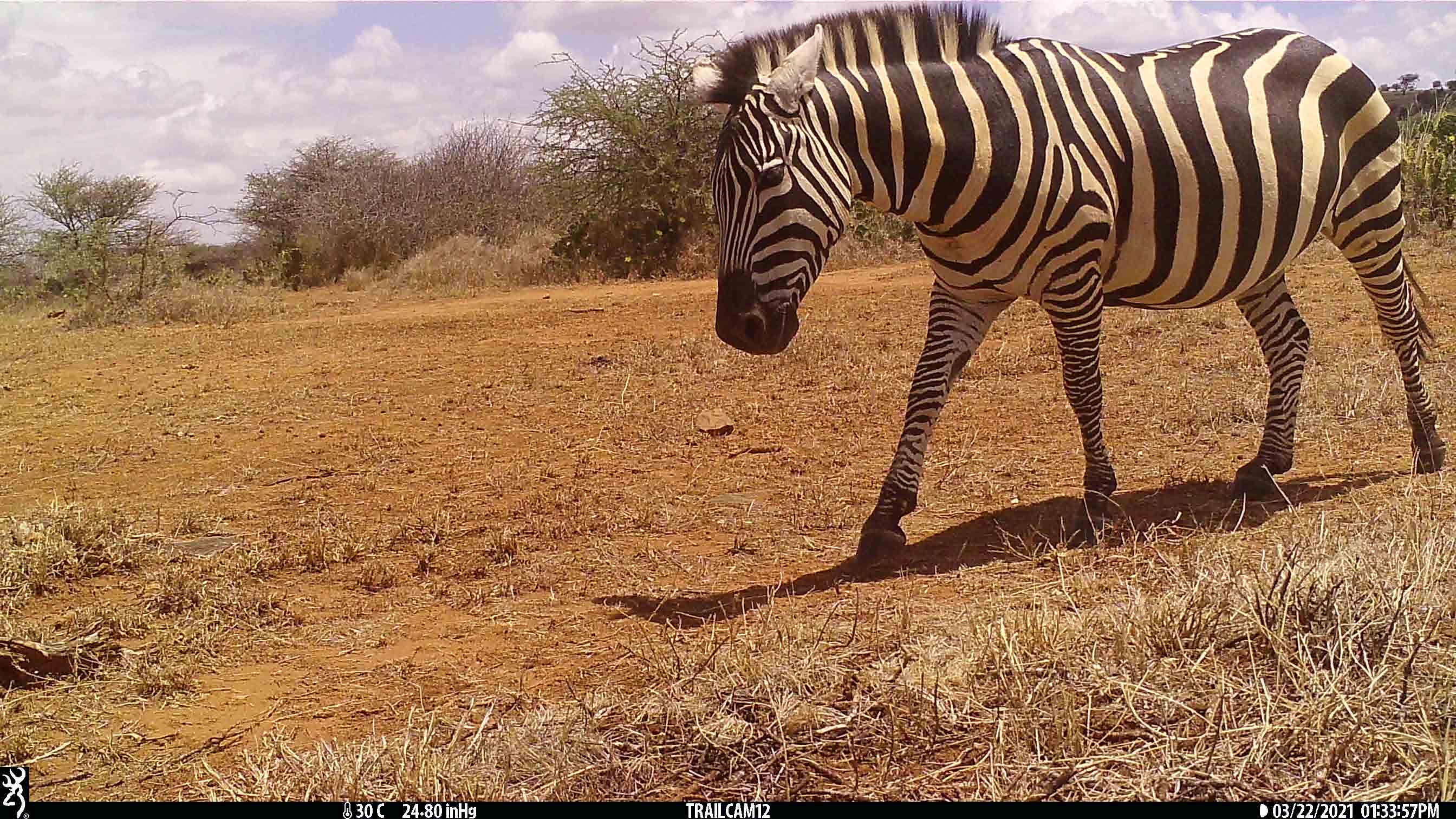


**C)**


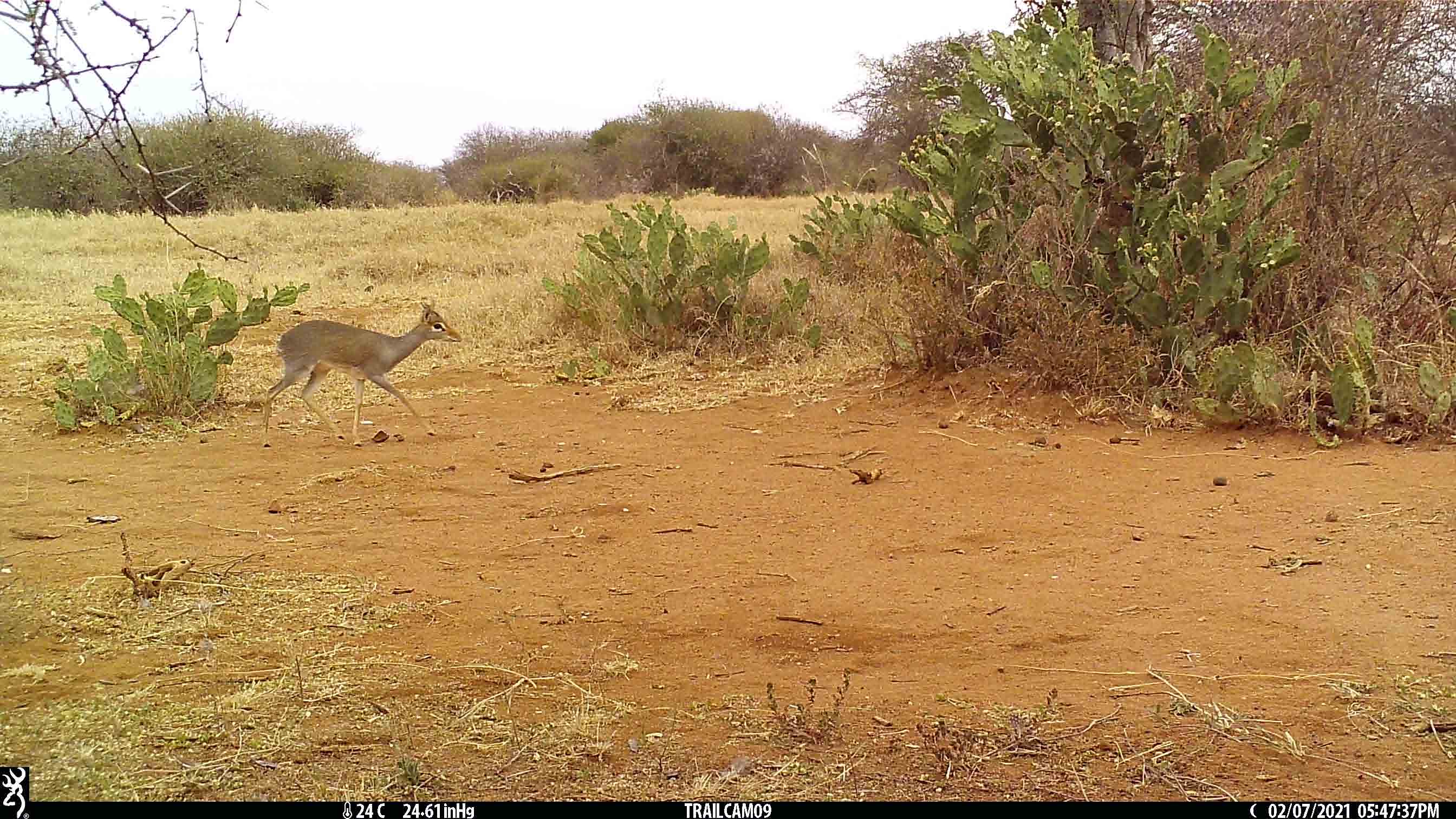


**D)**


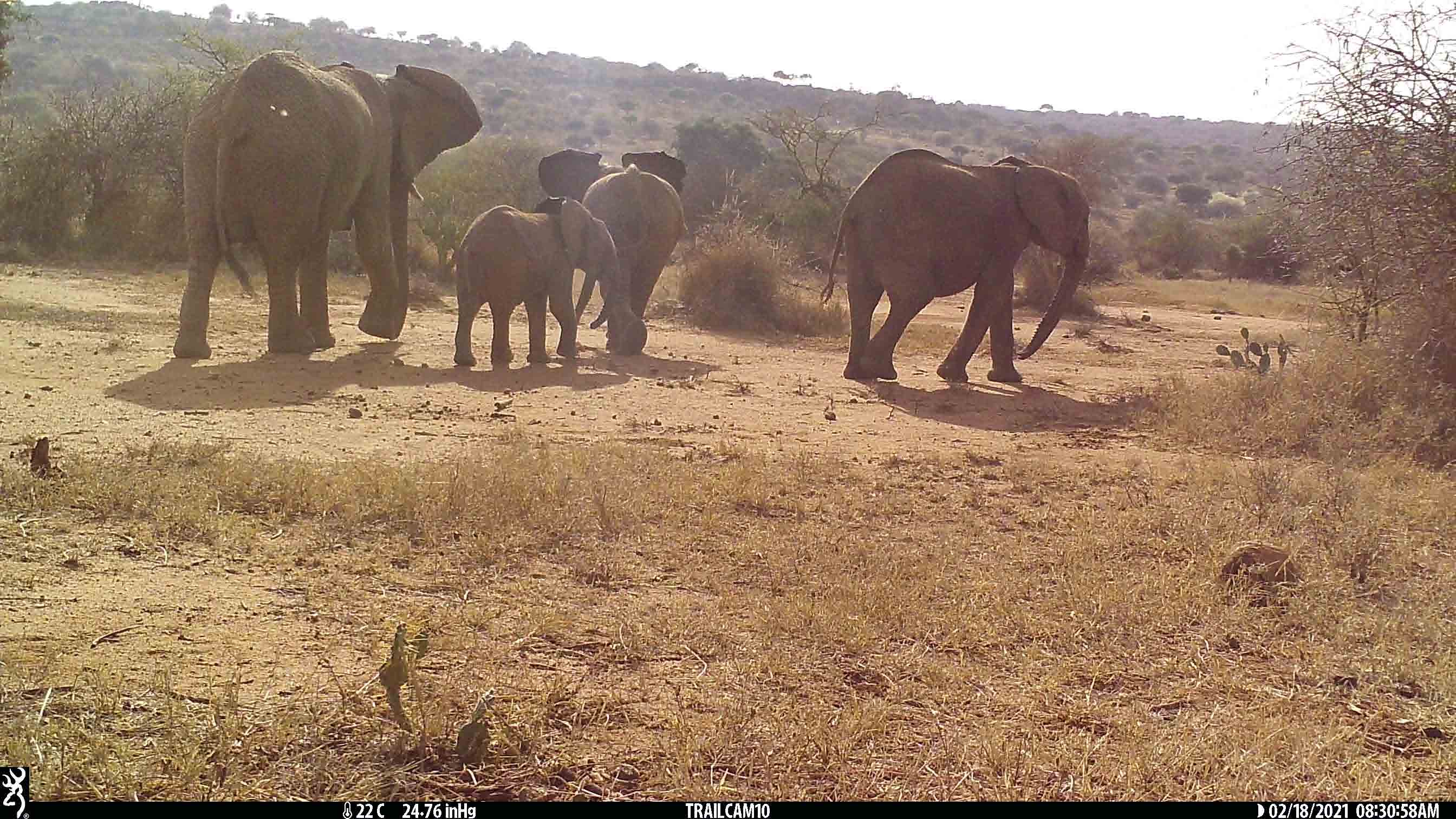


**Figure S1.** Camera trap photos from sites illustrating different quantities of site-level and grid square-level *Opuntia*: **A)** high site-level, high grid square-level, **B)** low site-level, high grid square-level, **C)** high site-level, low grid square-level, and **D)** low site-level, low grid square-level.

**
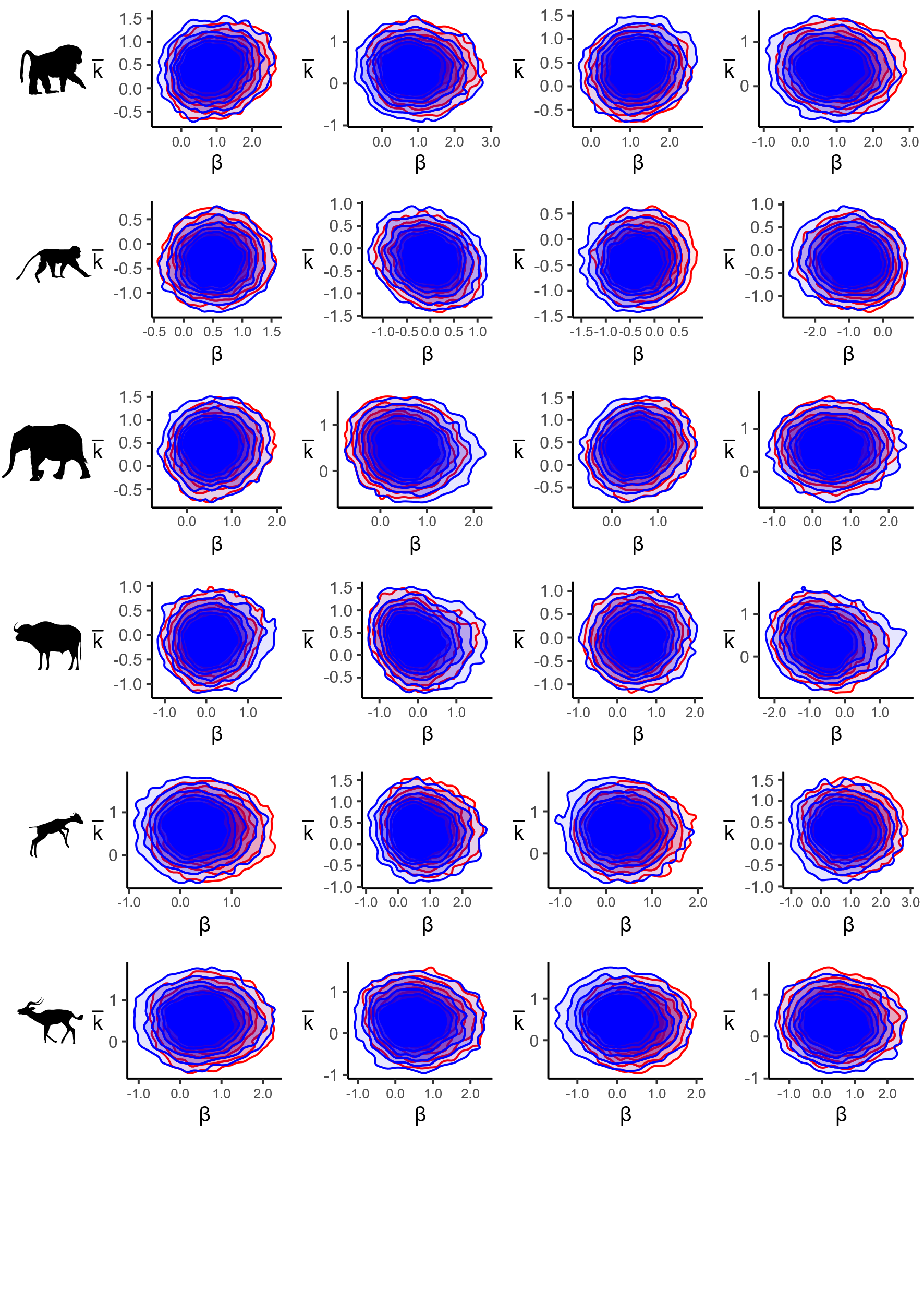
**

**
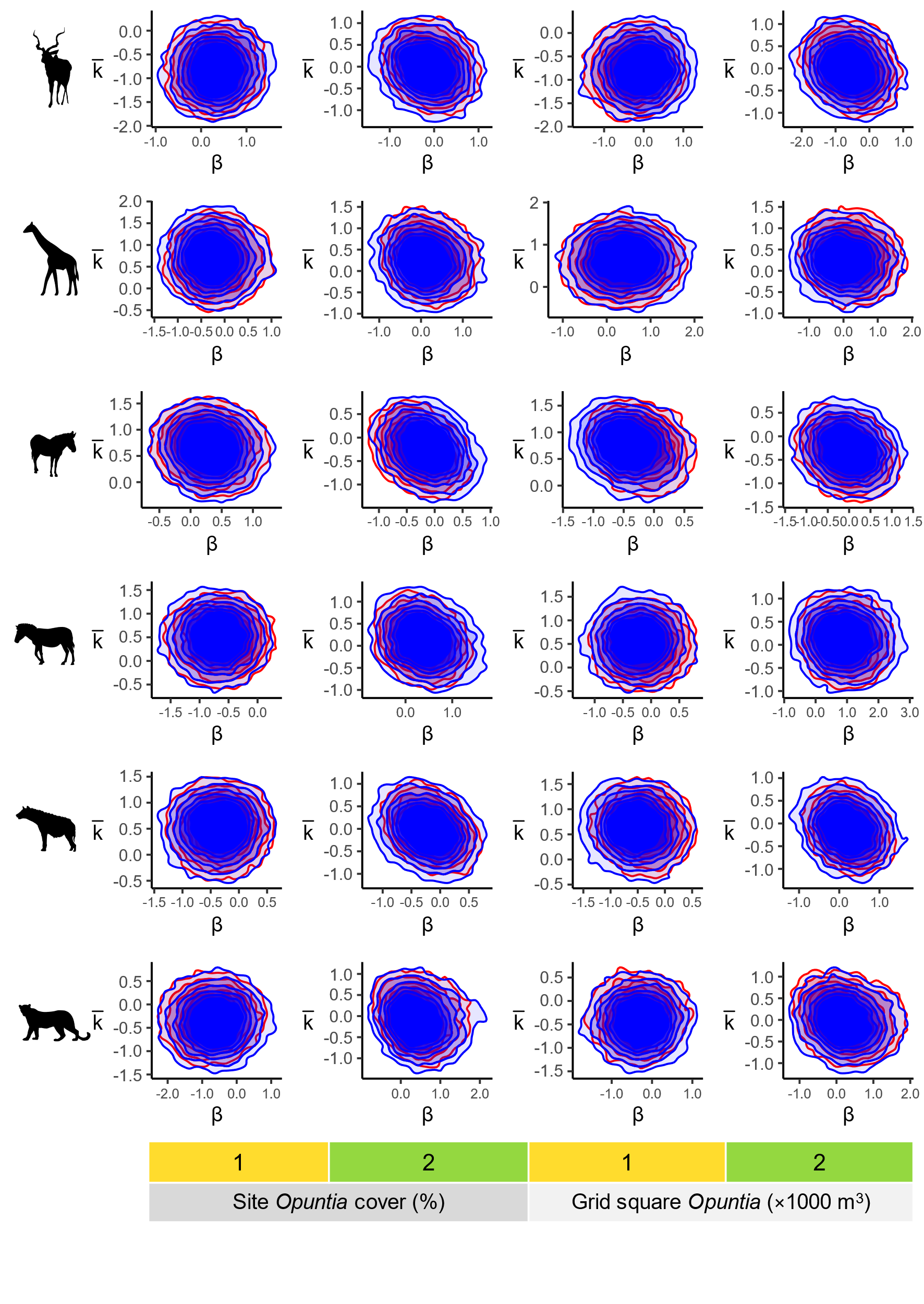
**

**Figure S2.** Kernel density contours for posterior distributions of the intercept ($\bar{k}$) and effect of *Opuntia* (β) for occupancy models which include (red) and omit (blue) measures of the native plant community (see Fig. 2 main text). The high degree of overlap between the distributions for these two models indicates that the inferred impact of *Opuntia* on occupancy was similar regardless of whether native plant variables were included. Contour bands represent (from outside) 95%, 89%, 80%, 70%, 60%, and 50% density regions. Rows represent mammal species (from top: olive baboon, vervet monkey, elephant, buffalo, dik-dik, impala, kudu, giraffe, Grevy’s zebra, plains zebra, spotted hyena, leopard). Columns indicate combinations of season (1 = January-April, 2 = October-November) and spatial scale of *Opuntia* covariate (site-level % cover, grid square-level volume).

**
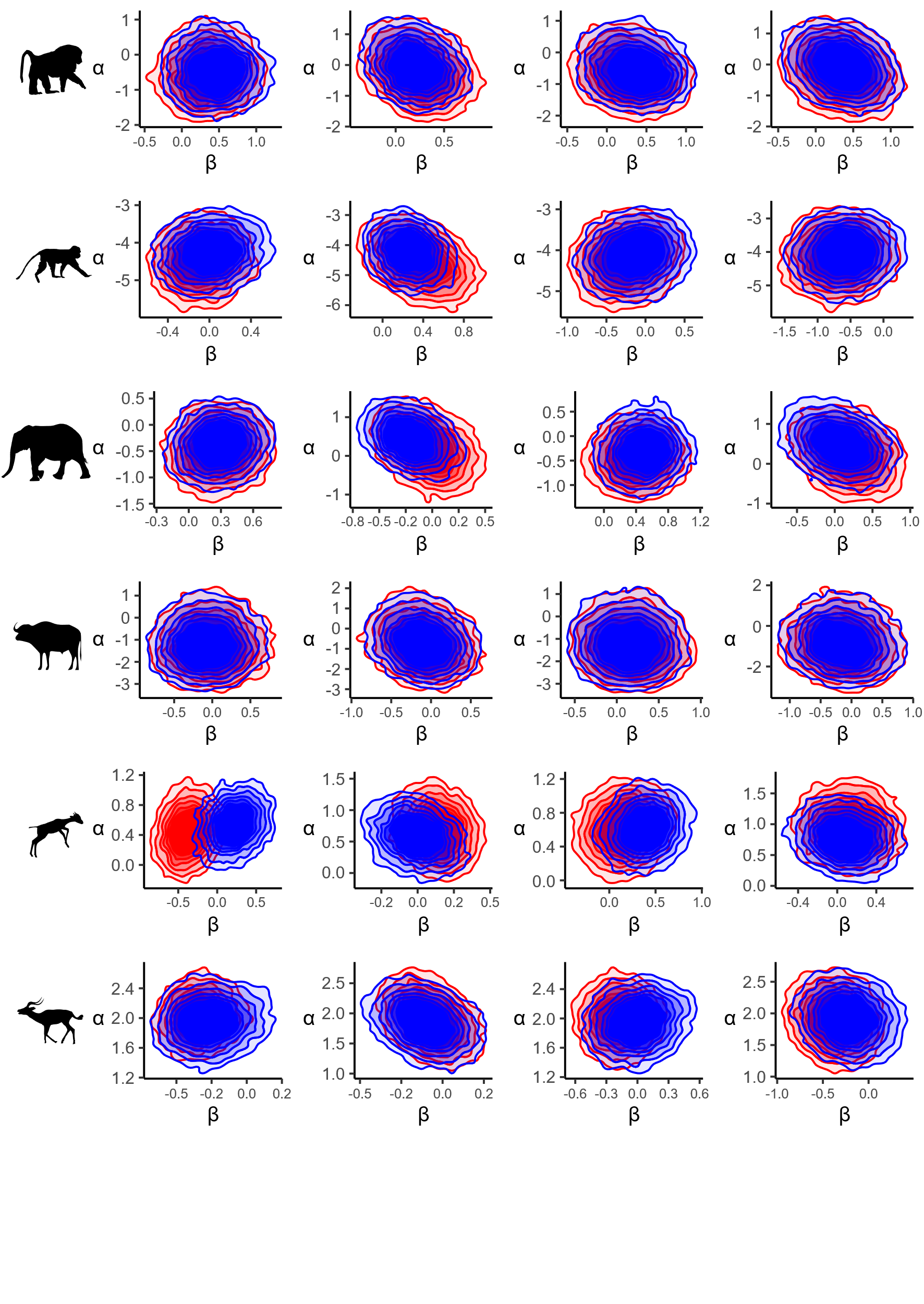
**

**
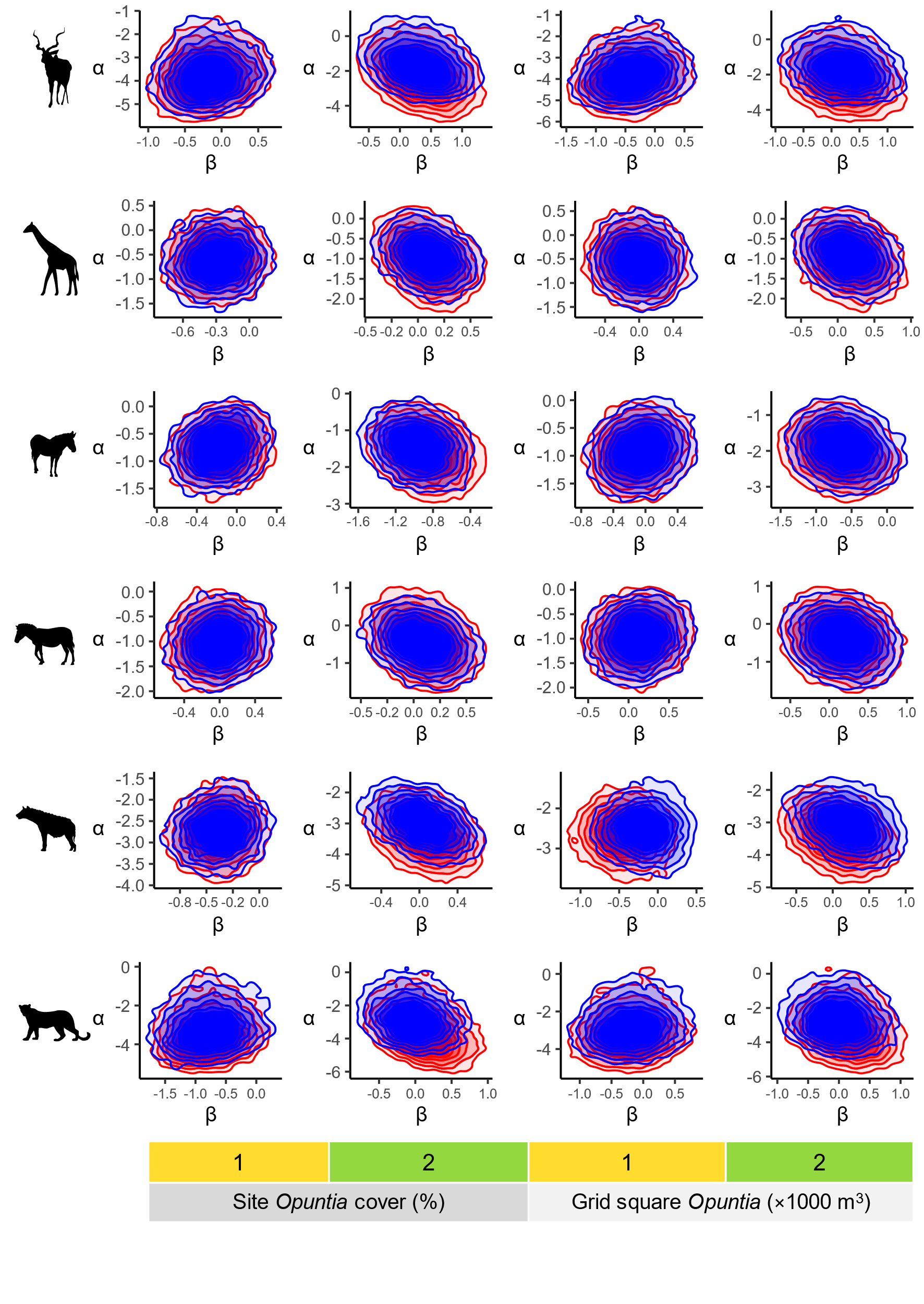
Figure S3.** Kernel density contours for posterior distributions of the intercept ($\bar{k}$) and effect of *Opuntia* (β) for total number of detection models which include (red) and omit (blue) measures of the native plant community (see Fig. 2 main text). The high degree of overlap between the distributions for these two models indicates that, for most species, the inferred impact of *Opuntia* on total number of detections per day was similar regardless of whether native plant variables were included. Notably, the distributions for the effect of site-level *Opuntia* cover on dik-dik in season 1 displayed relatively little overlap, suggesting that the effects of *Opuntia* may be partially mediated by effects on native vegetation cover in this case. Contour bands represent (from outside) 95%, 89%, 80%, 70%, 60%, and 50% density regions. Rows represent mammal species (from top: olive baboon, vervet monkey, elephant, buffalo, dik-dik, impala, kudu, giraffe, Grevy’s zebra, plains zebra, spotted hyena, leopard). Columns indicate combinations of season (1 = January-April, 2 = October-November) and spatial scale of *Opuntia* covariate (site-level % cover, grid square-level volume).

**
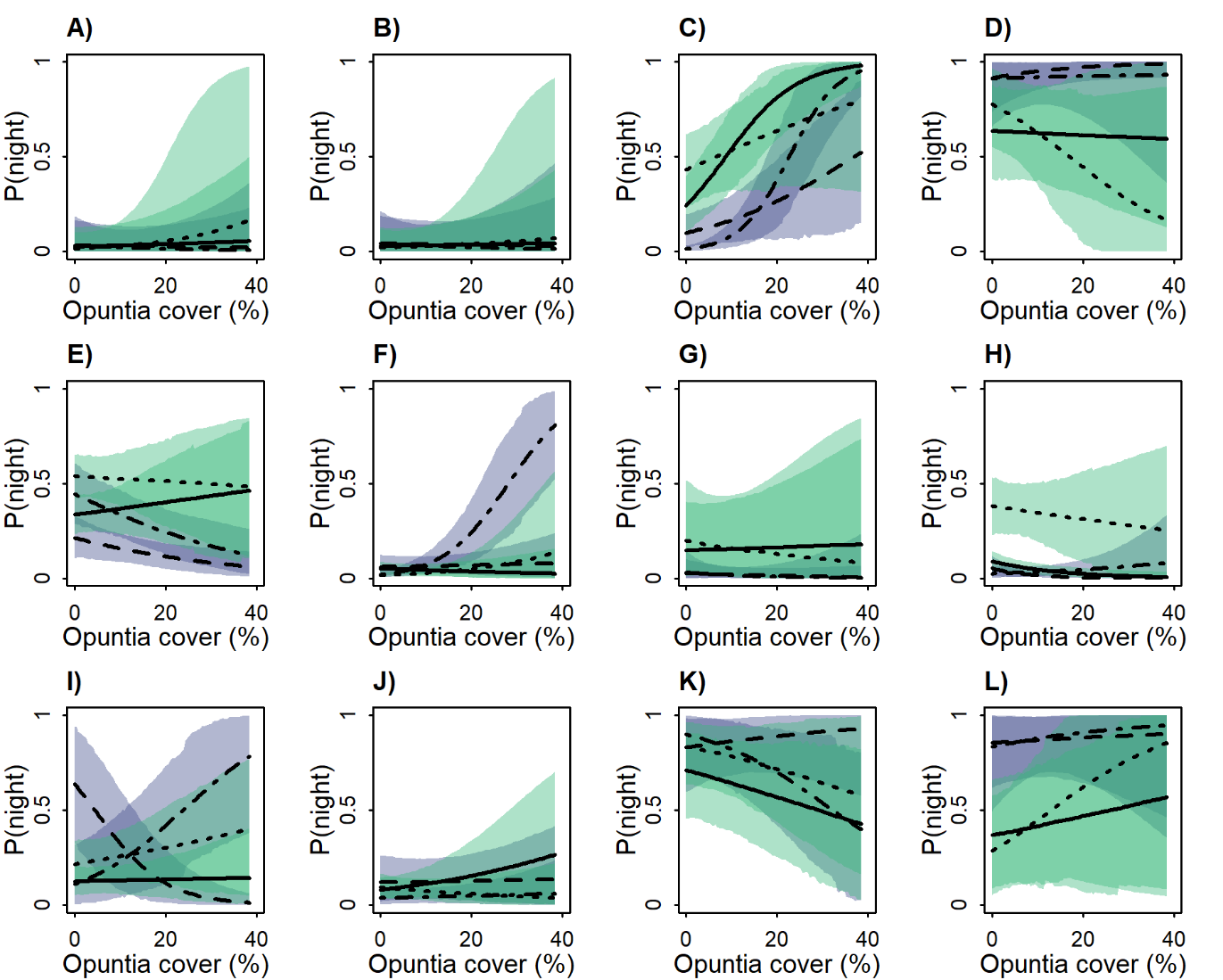
**


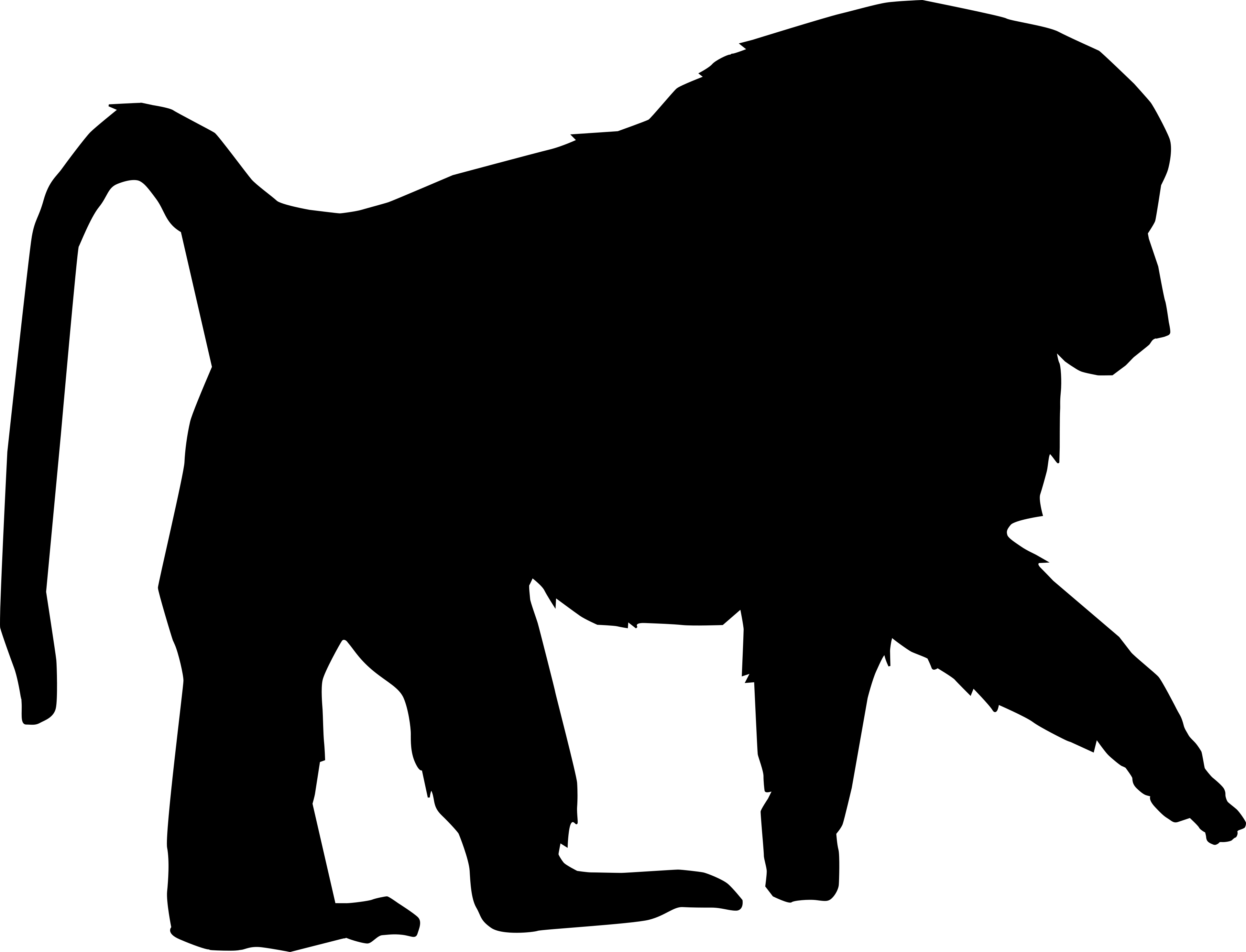

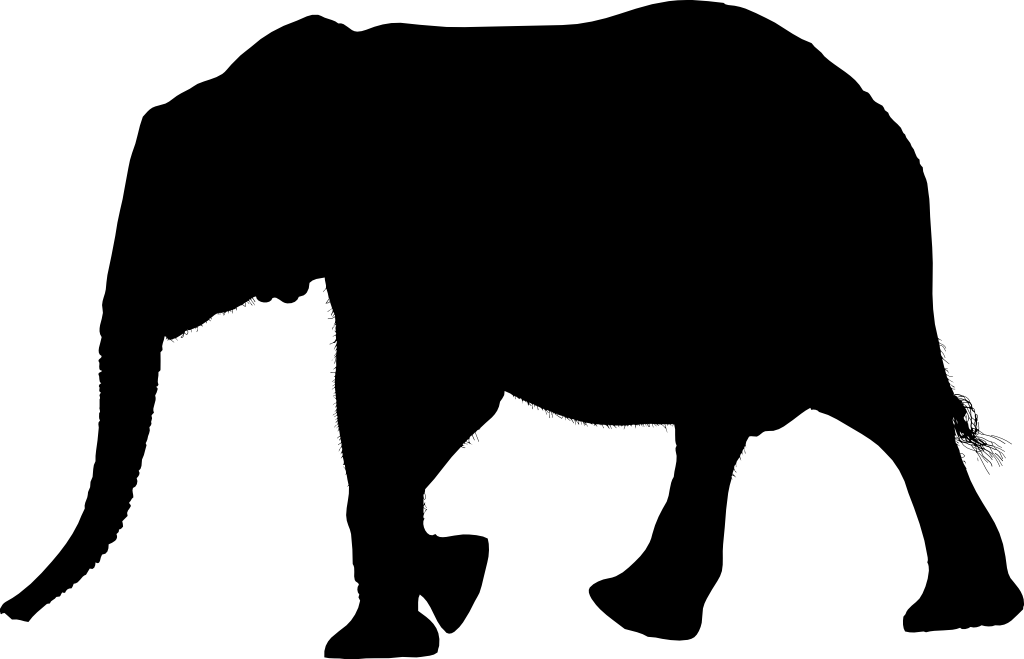

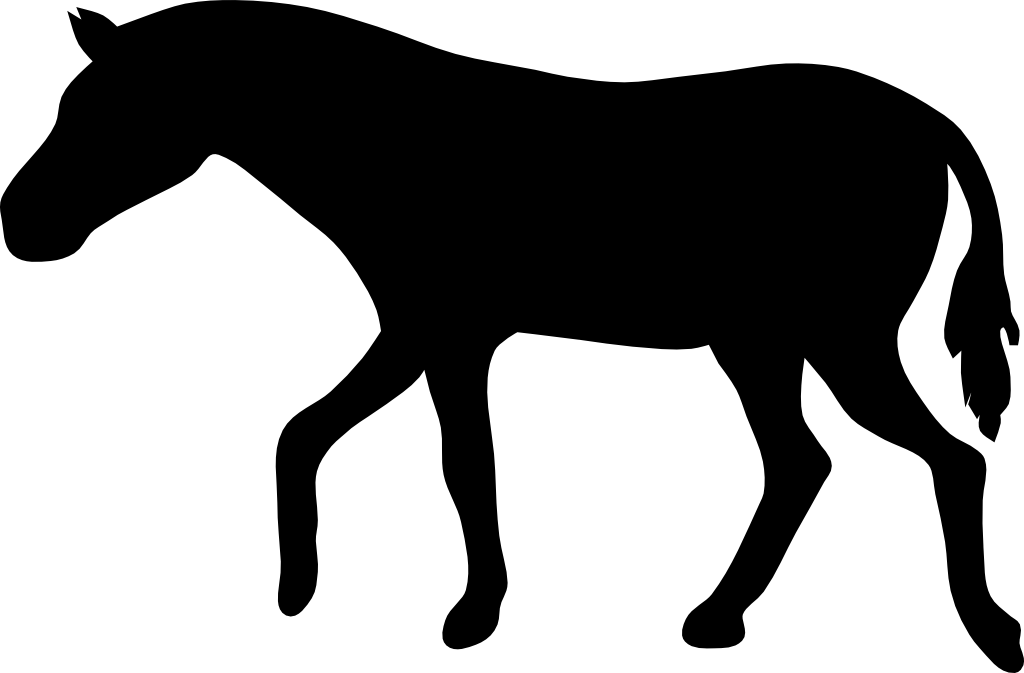

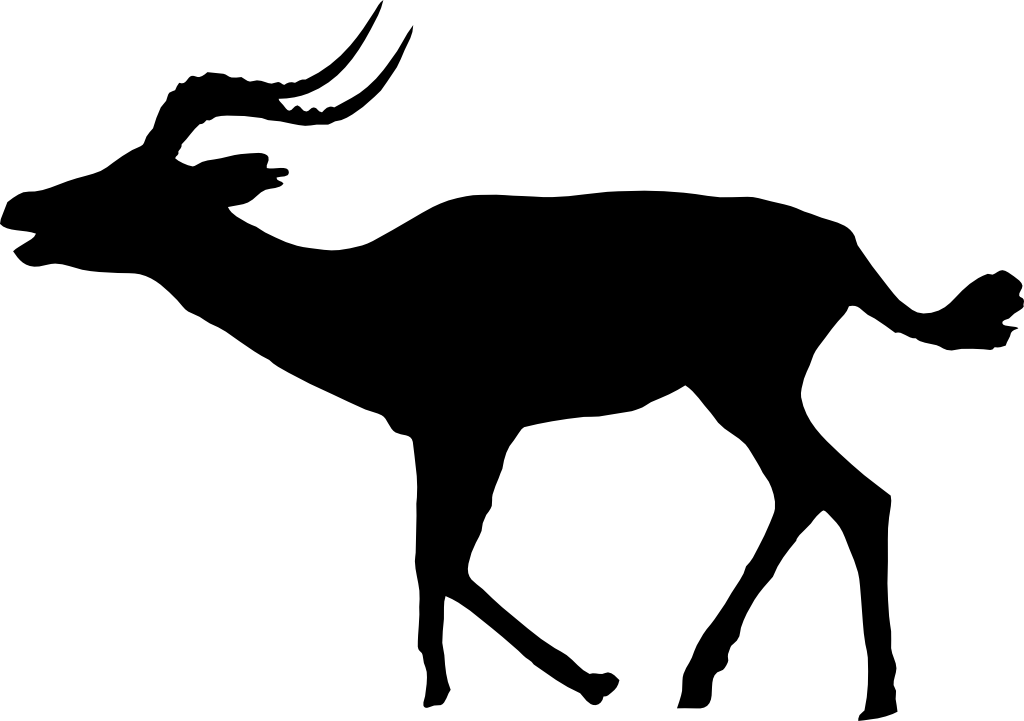

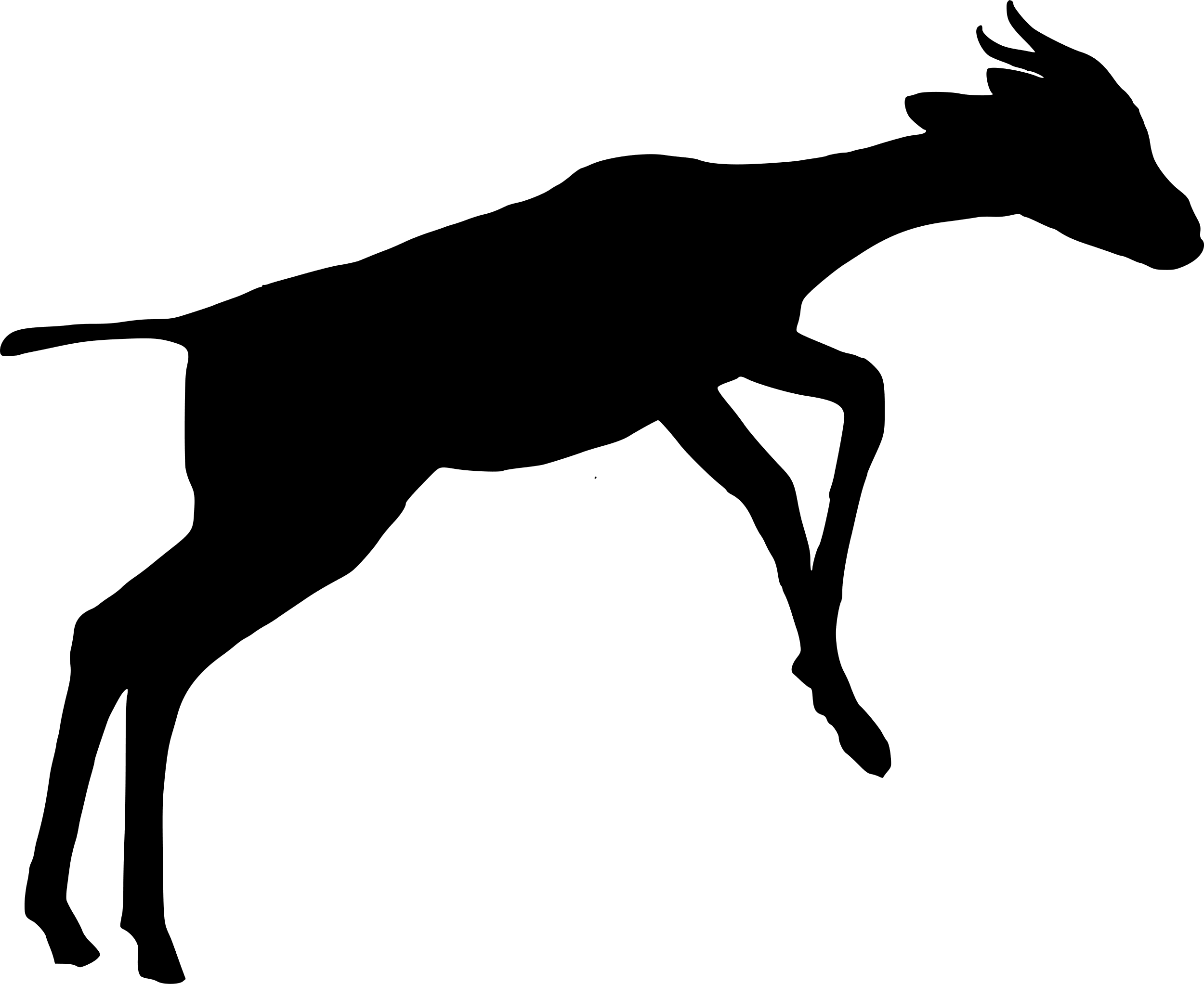

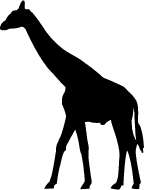

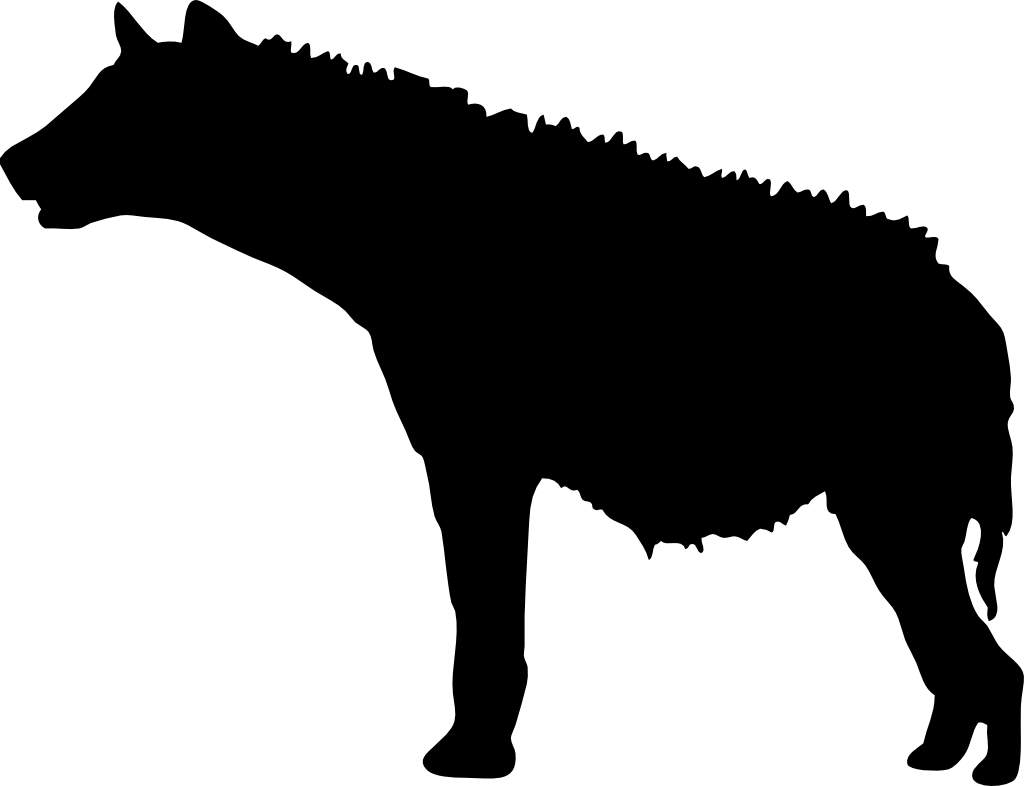

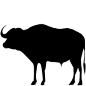

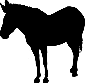

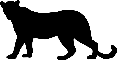


**Figure S4.** Marginal total effect of site-level *Opuntia* percentage cover on the proportion of detections occurring at night for: **A)** olive baboon, **B)** vervet monkey, **C)** elephant, **D)** buffalo, **E)** dik-dik, **F)** impala, **G)** kudu, **H)** giraffe, **I)** Grevy’s zebra, **J)** plains zebra, **K)** spotted hyena, and **L)** leopard. The models assume that *Opuntia* does not indirectly affect occupancy through altering the composition of the native plant community; for model structure, see Figure 2 in main text. Shaded areas represent 89 compatibility intervals for the January-April (light green) and October-November (purple) seasons. Black lines indicate posterior median marginal effects for January-April under a new moon (―) and full moon (‧ ‧ ‧), and October-November under a new moon (– – –) and full moon (– ‧ –).

**
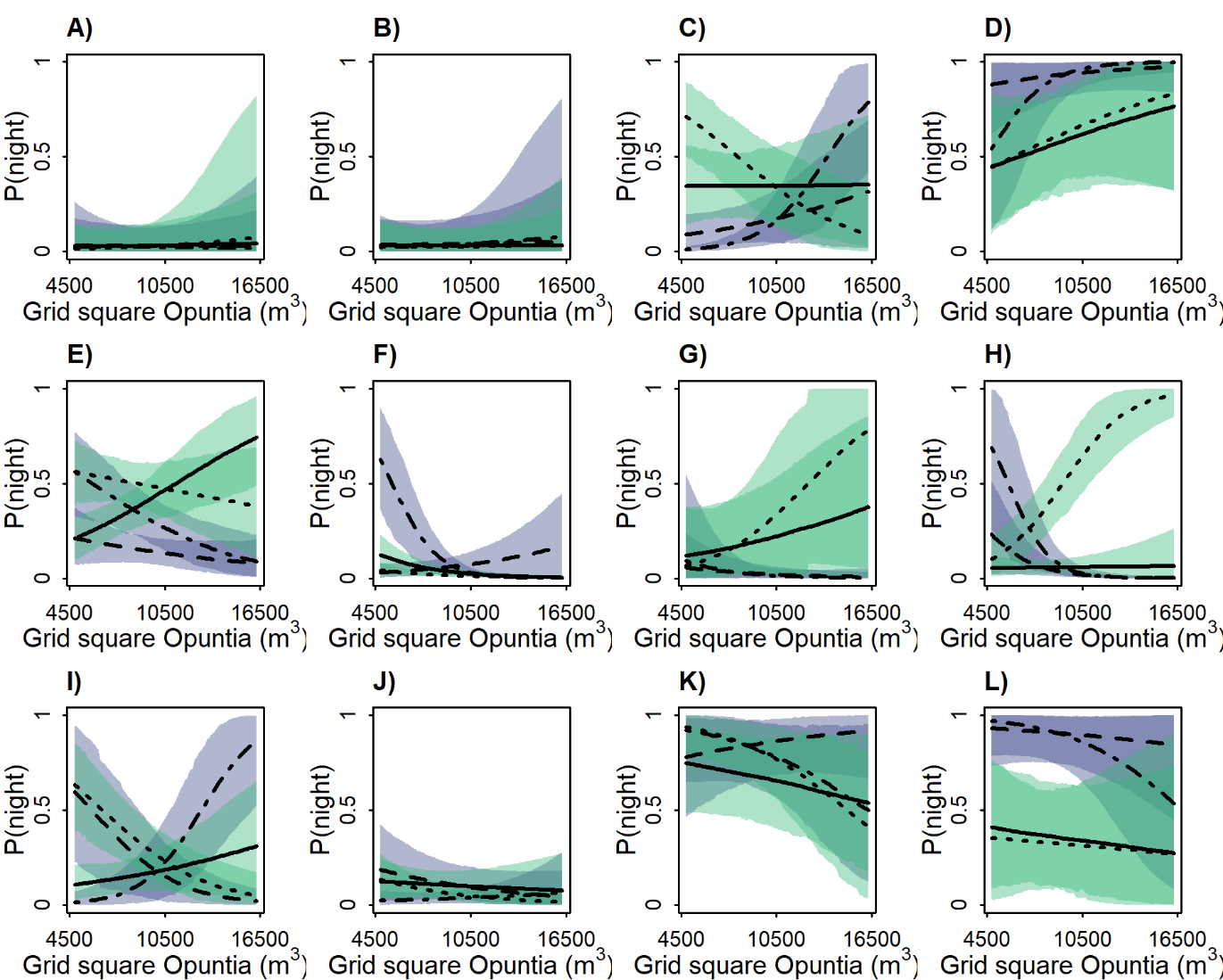
**


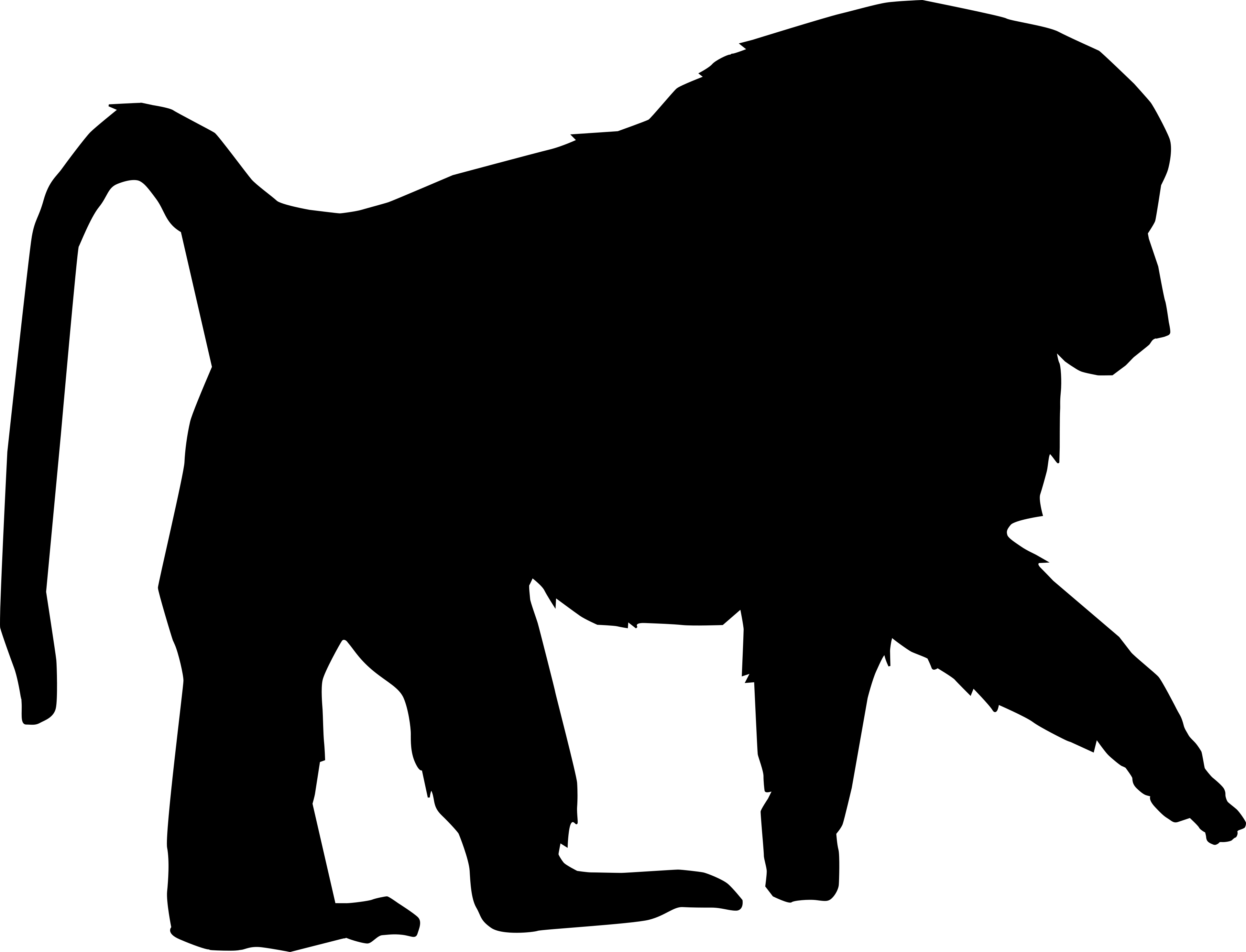

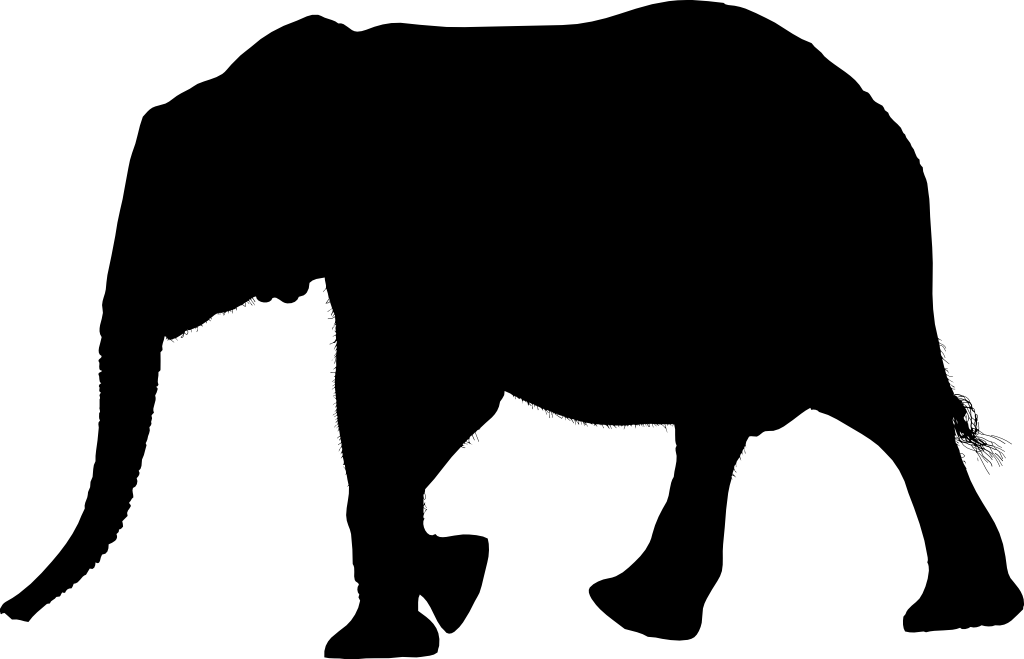

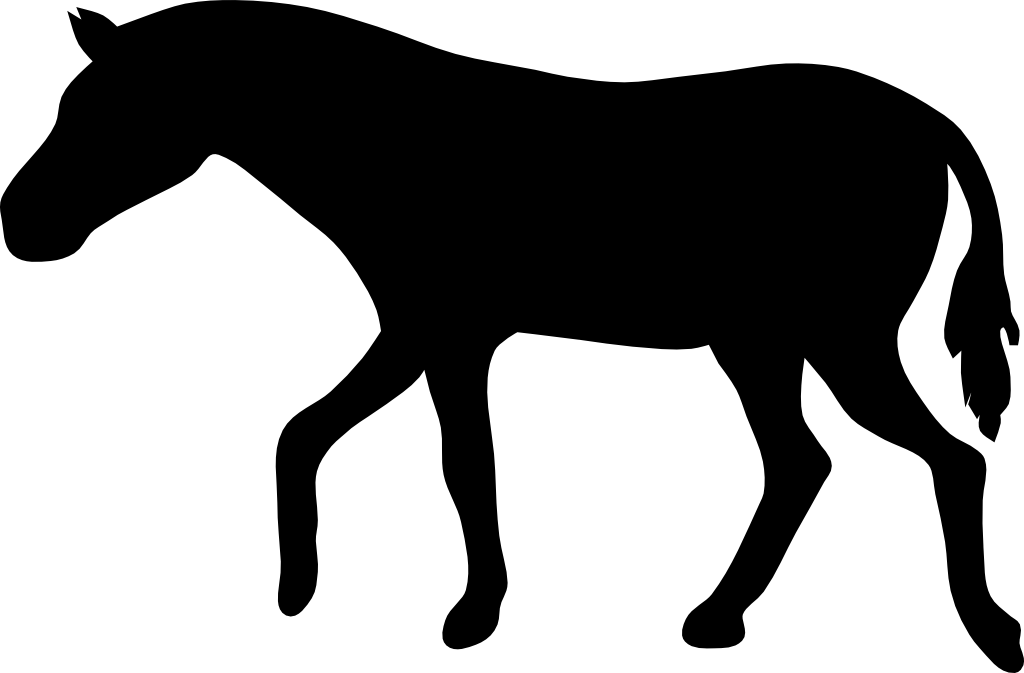

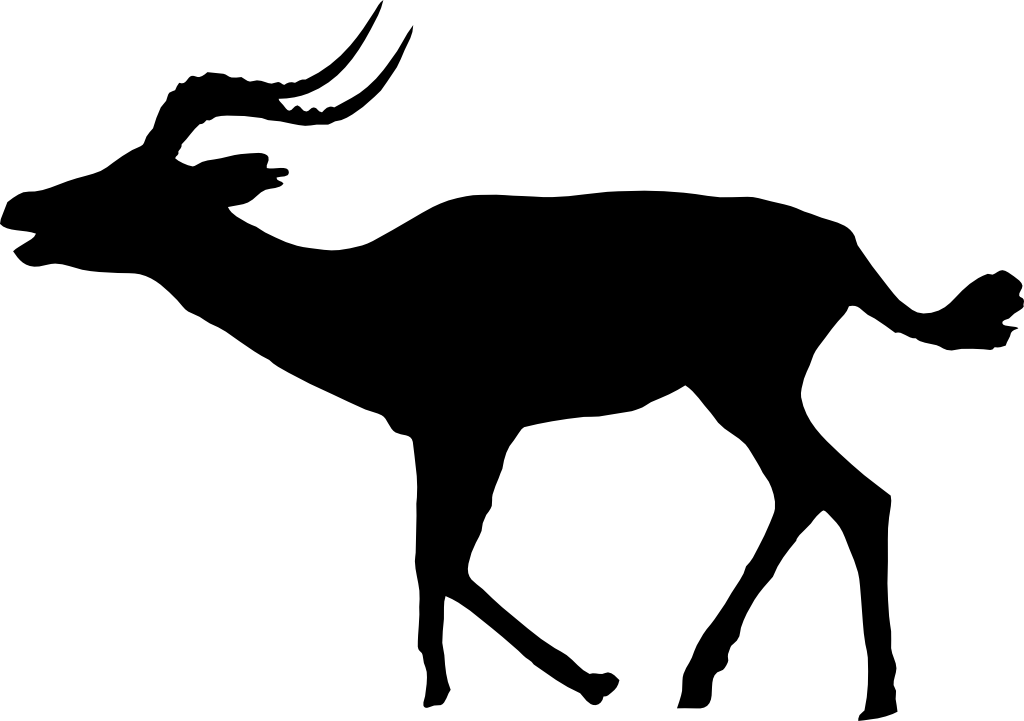

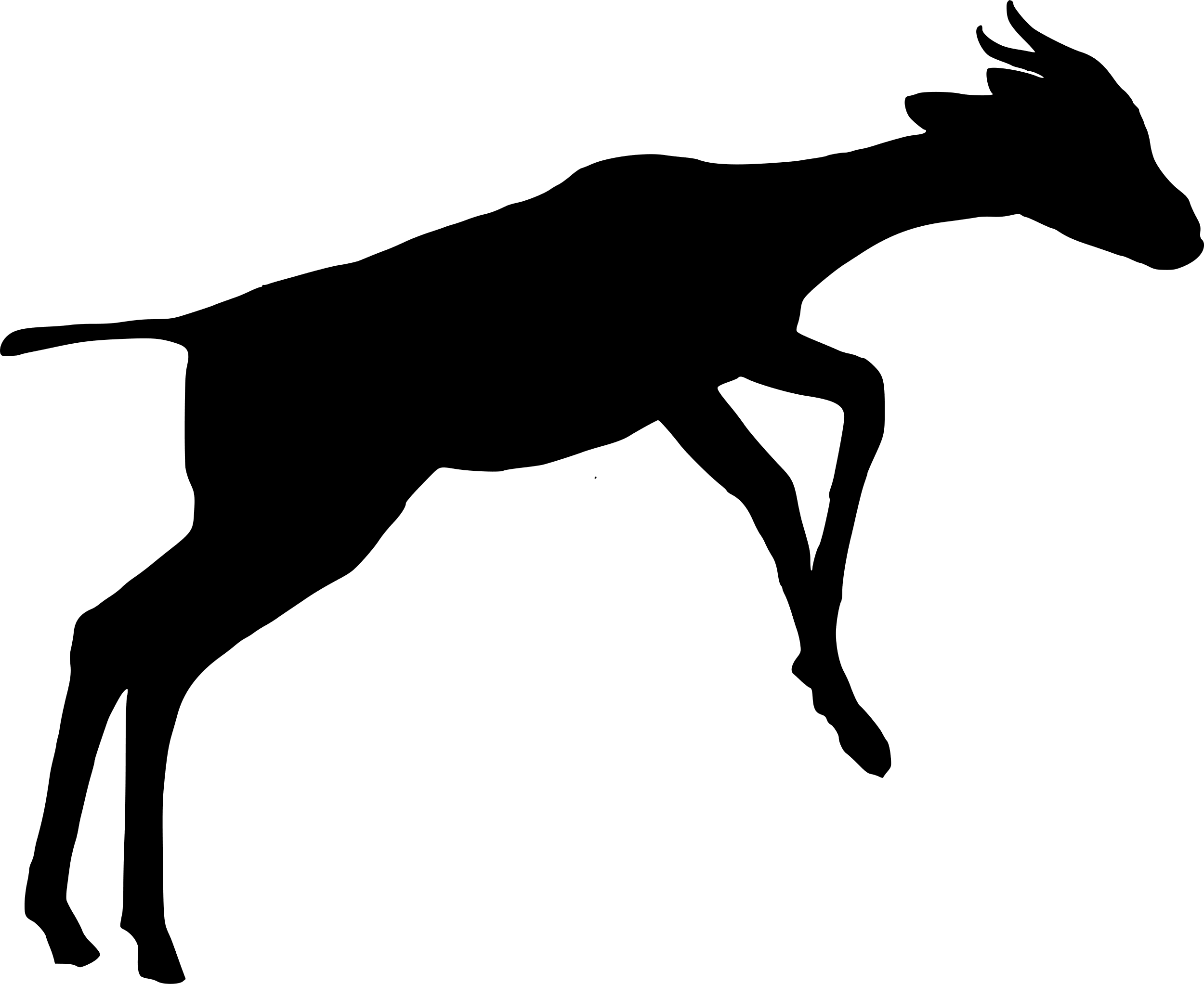

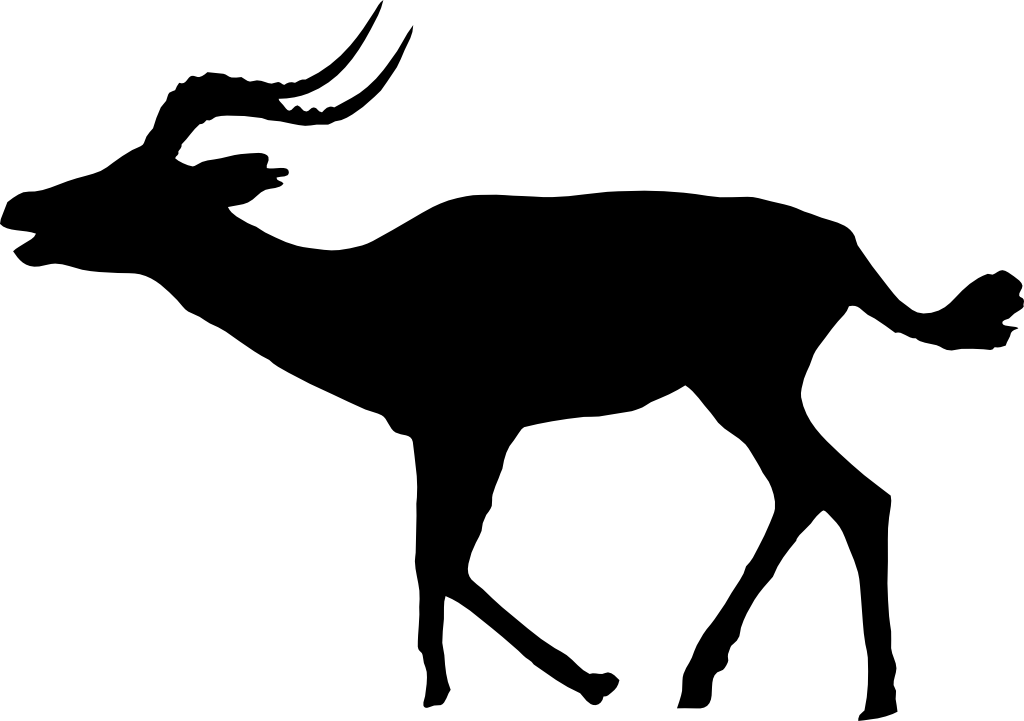

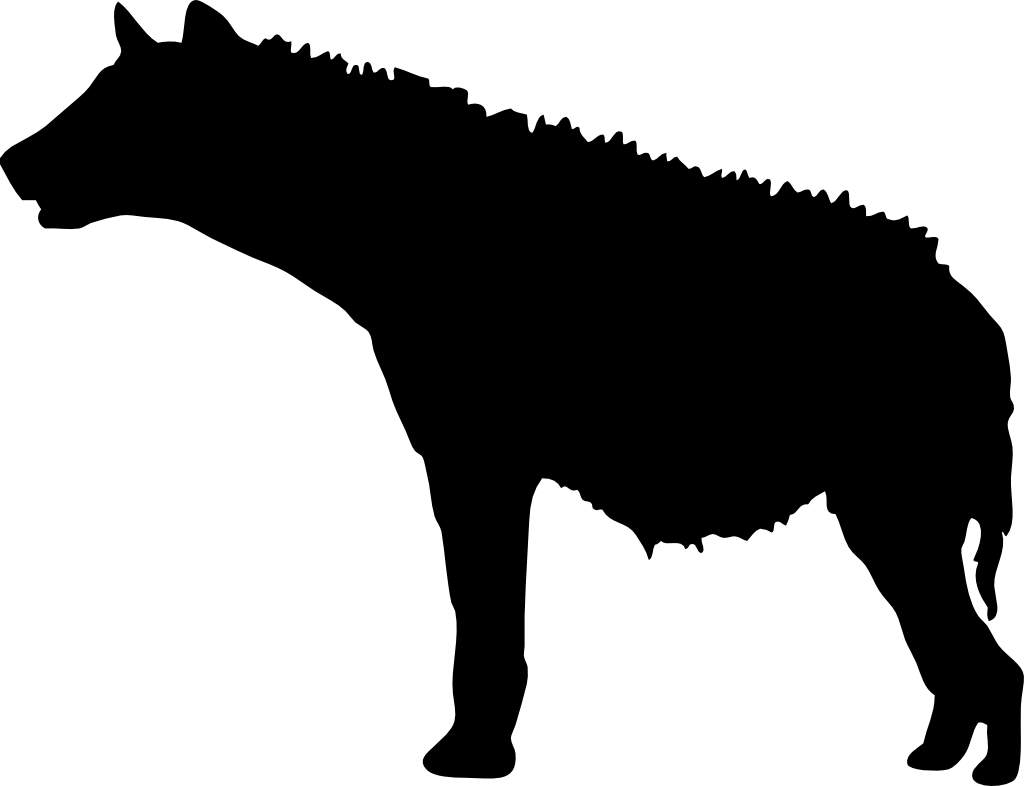

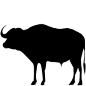

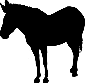

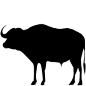


**Figure S5.** Marginal total effect of grid square-level *Opuntia* volume on the proportion of detections occurring at night for: **A)** olive baboon, **B)** vervet monkey, **C)** elephant, **D)** buffalo, **E)** dik-dik, **F)** impala, **G)** kudu, **H)** giraffe, **I)** Grevy’s zebra, **J)** plains zebra, **K)** spotted hyena, and **L)** leopard. The models assume that *Opuntia* does not indirectly affect occupancy through altering the composition of the native plant community; for model structure, see Figure 2 in main text. Shaded areas represent 89 compatibility intervals for the January-April (light green) and October-November (purple) seasons. Black lines indicate posterior median marginal effects for January-April under a new moon (―) and full moon (‧ ‧ ‧), and October-November under a new moon (– – –) and full moon (– ‧ –).

**
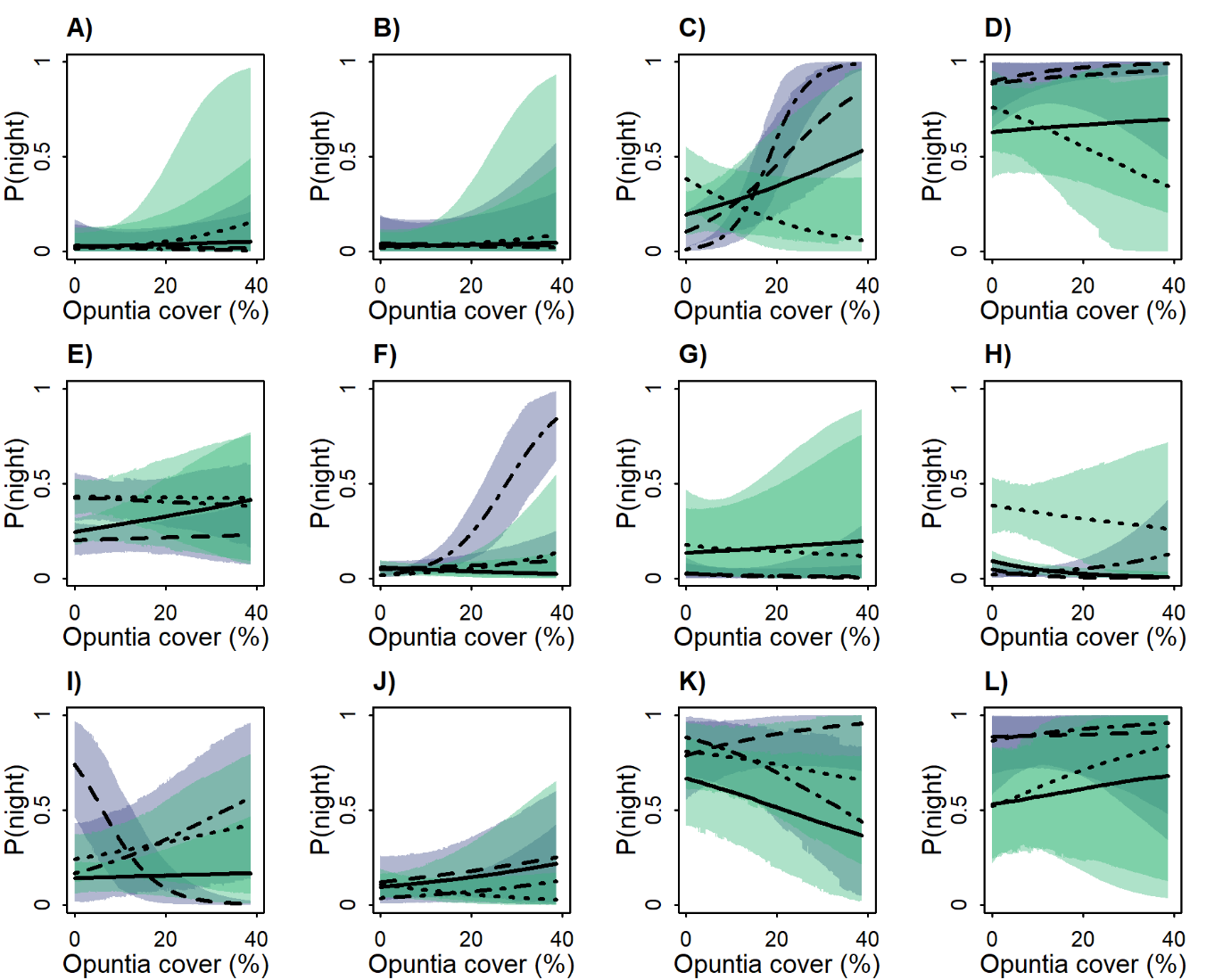
**


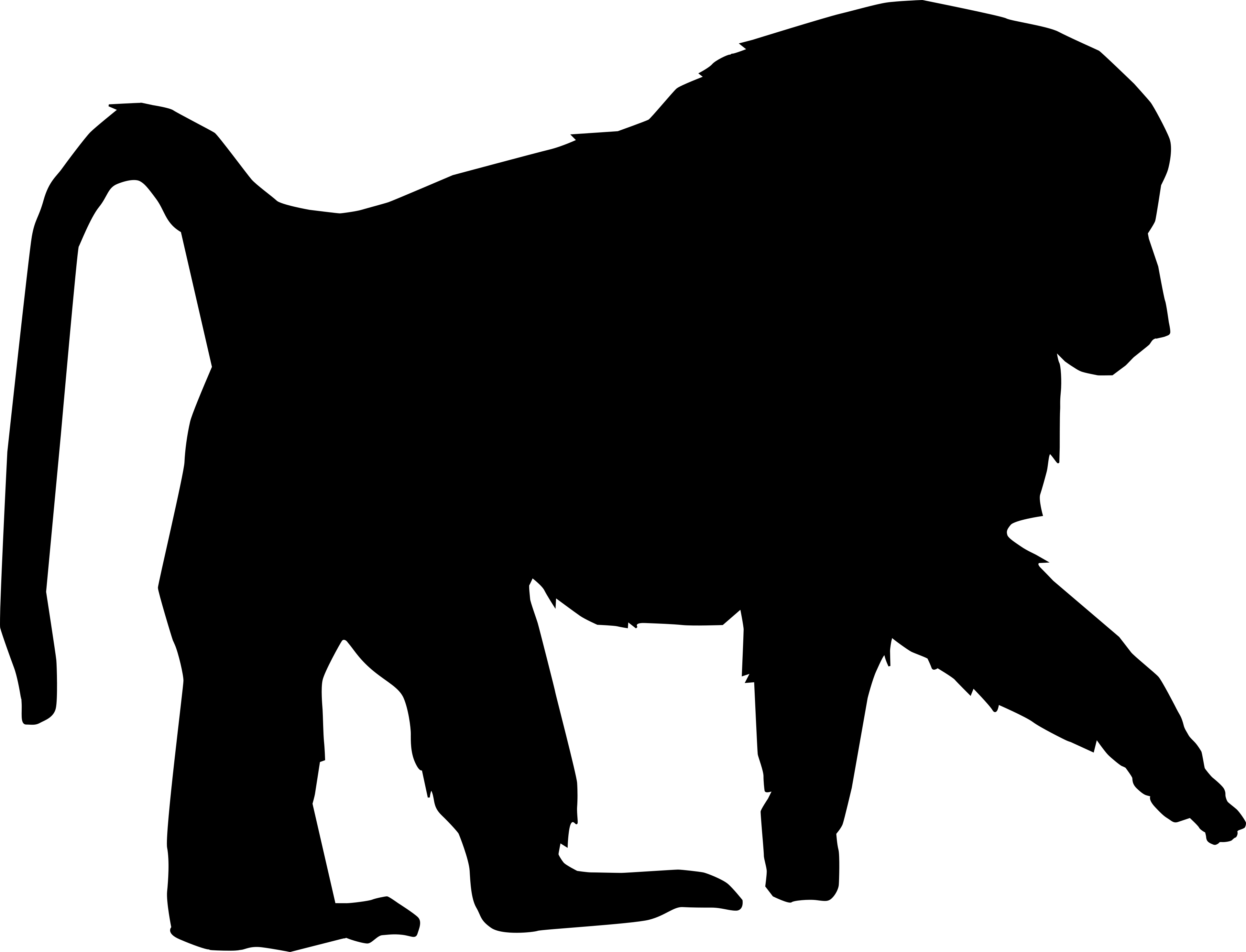

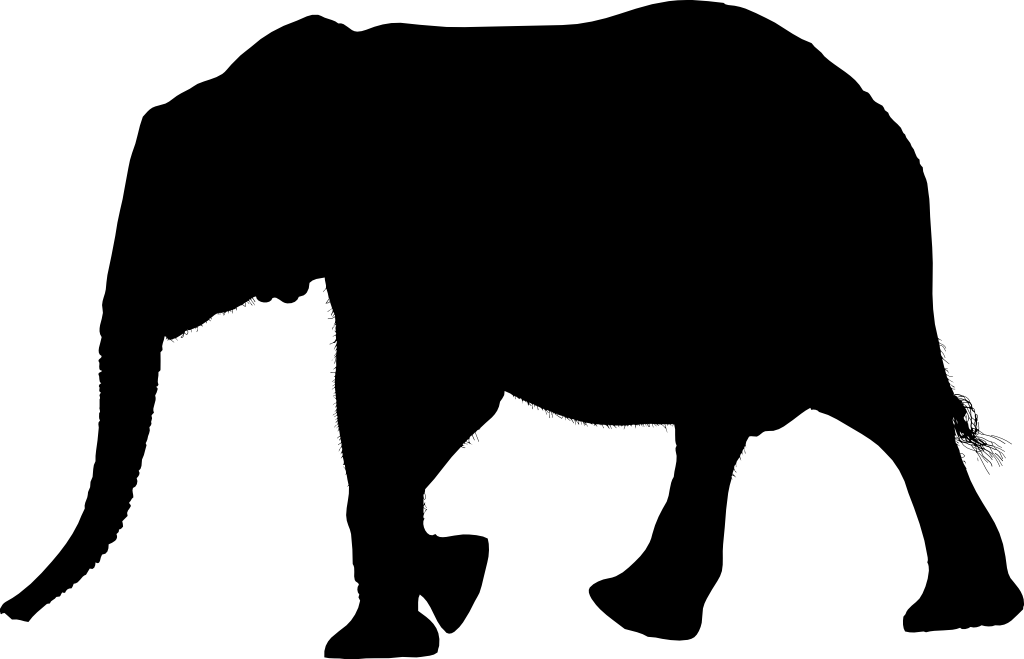

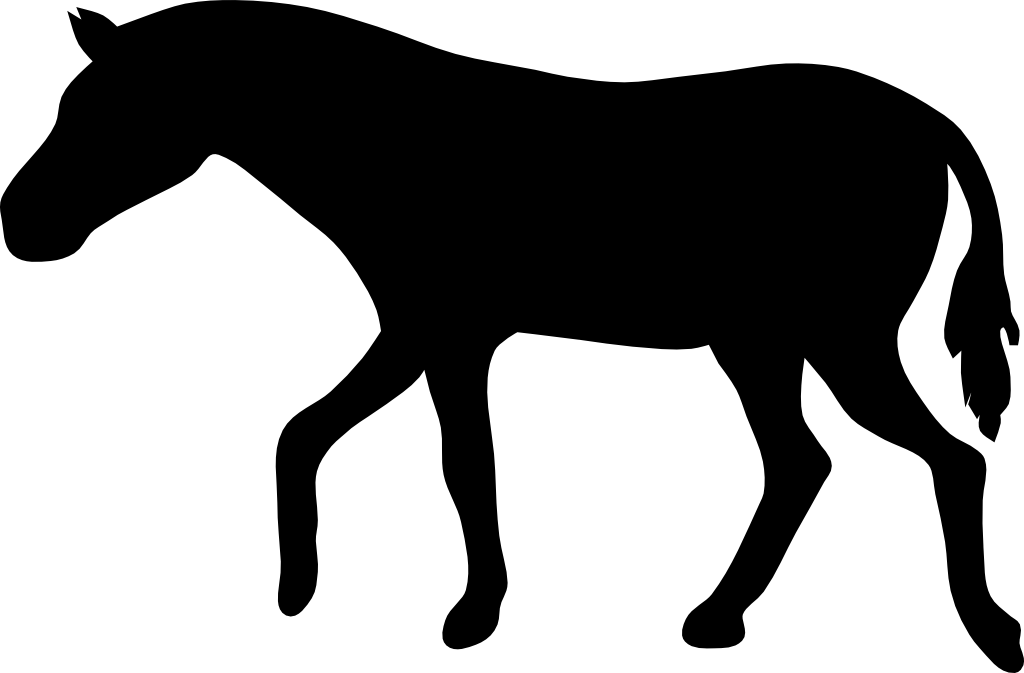

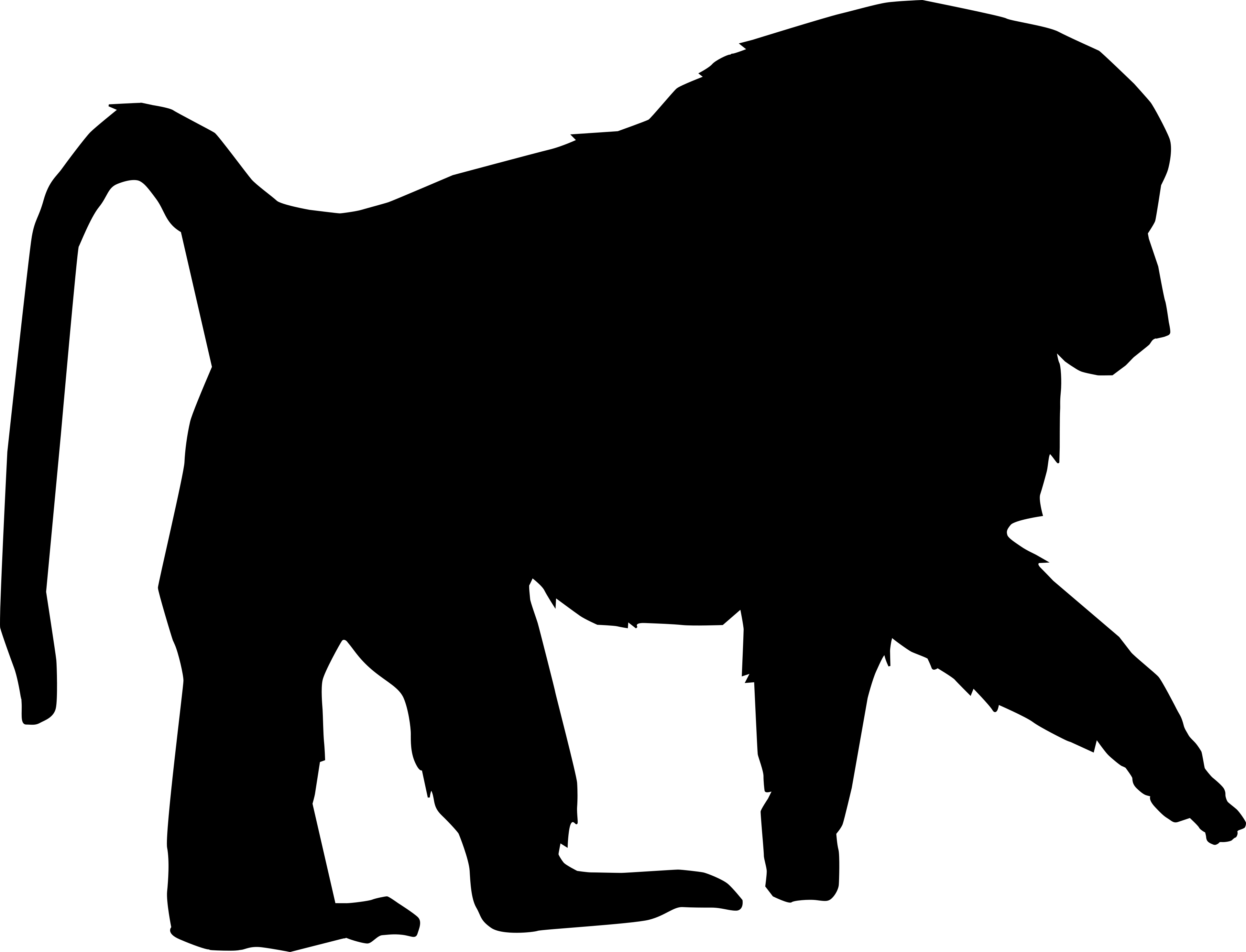

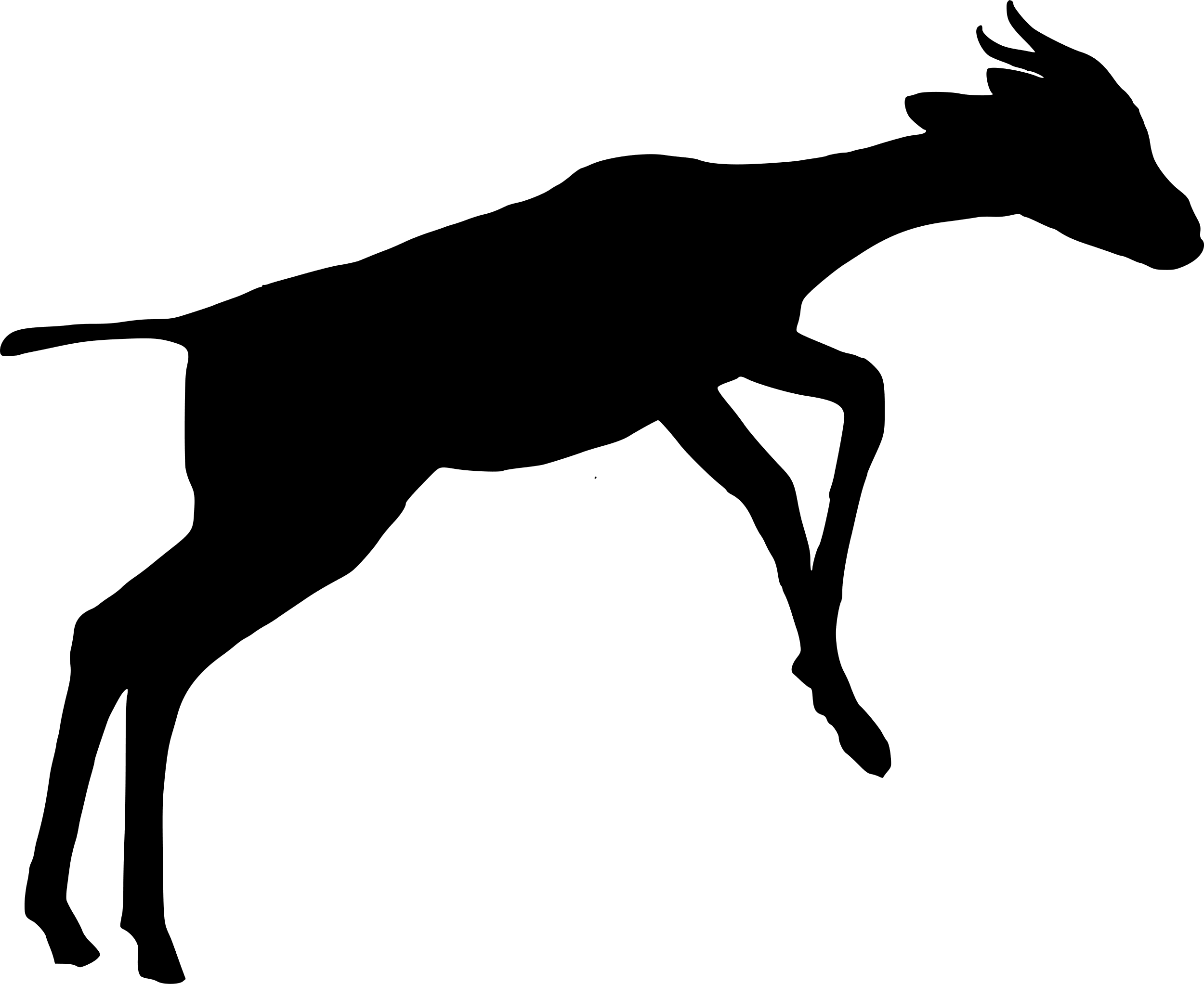

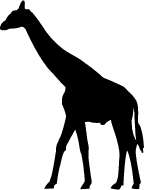

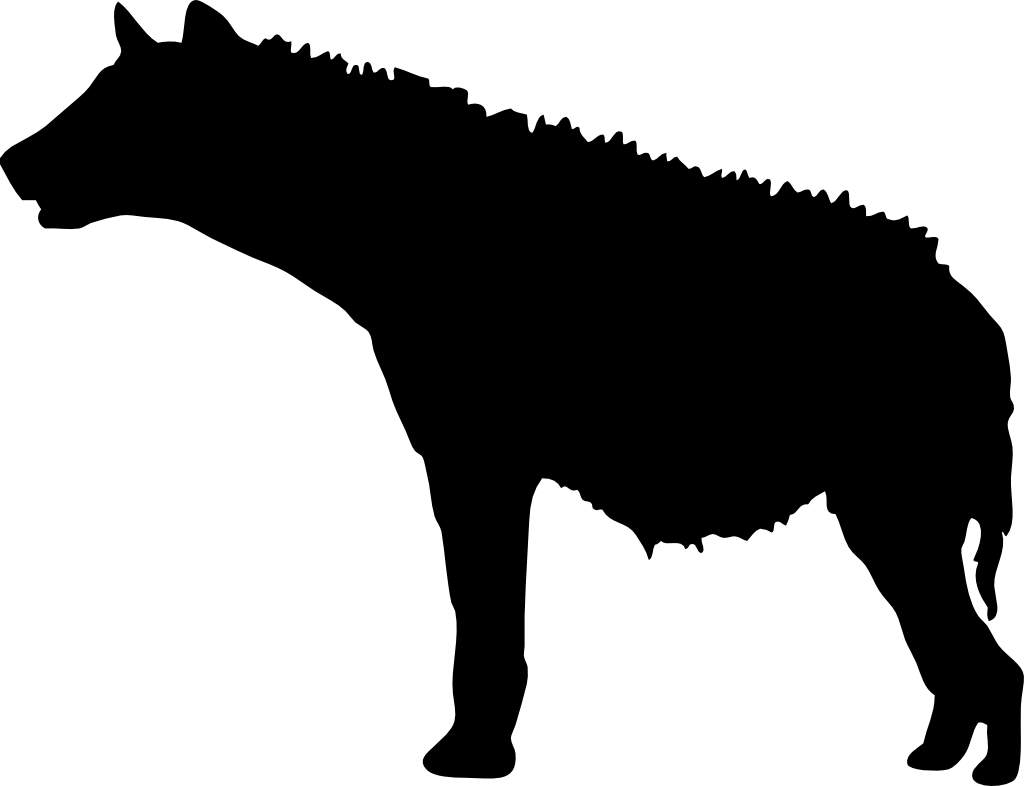

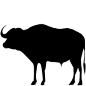

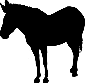

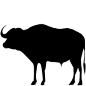


**Figure S6.** Marginal total effect of site-level *Opuntia* percentage cover on the proportion of detections occurring at night for: **A)** olive baboon, **B)** vervet monkey, **C)** elephant, **D)** buffalo, **E)** dik-dik, **F)** impala, **G)** kudu, **H)** giraffe, **I)** Grevy’s zebra, **J)** plains zebra, **K)** spotted hyena, and **L)** leopard. The models assume that *Opuntia* indirectly affects occupancy through altering the composition of the native plant community; for model structure, see Figure 2 in main text. Shaded areas represent 89 compatibility intervals for the January-April (light green) and October-November (purple) seasons. Black lines indicate posterior median marginal effects for January-April under a new moon (―) and full moon (‧ ‧ ‧), and October-November under a new moon (– – –) and full moon (– ‧ –).

**
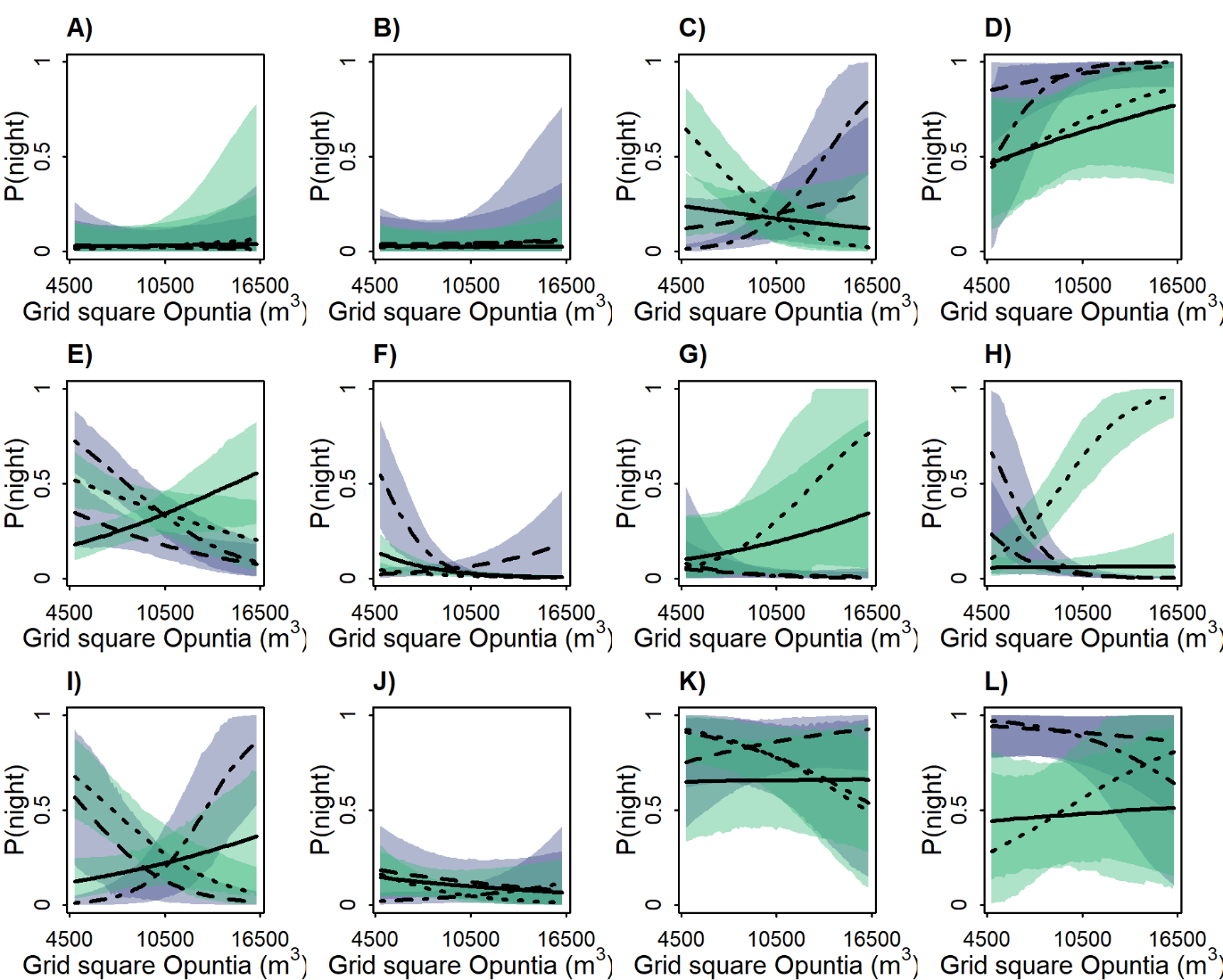
**


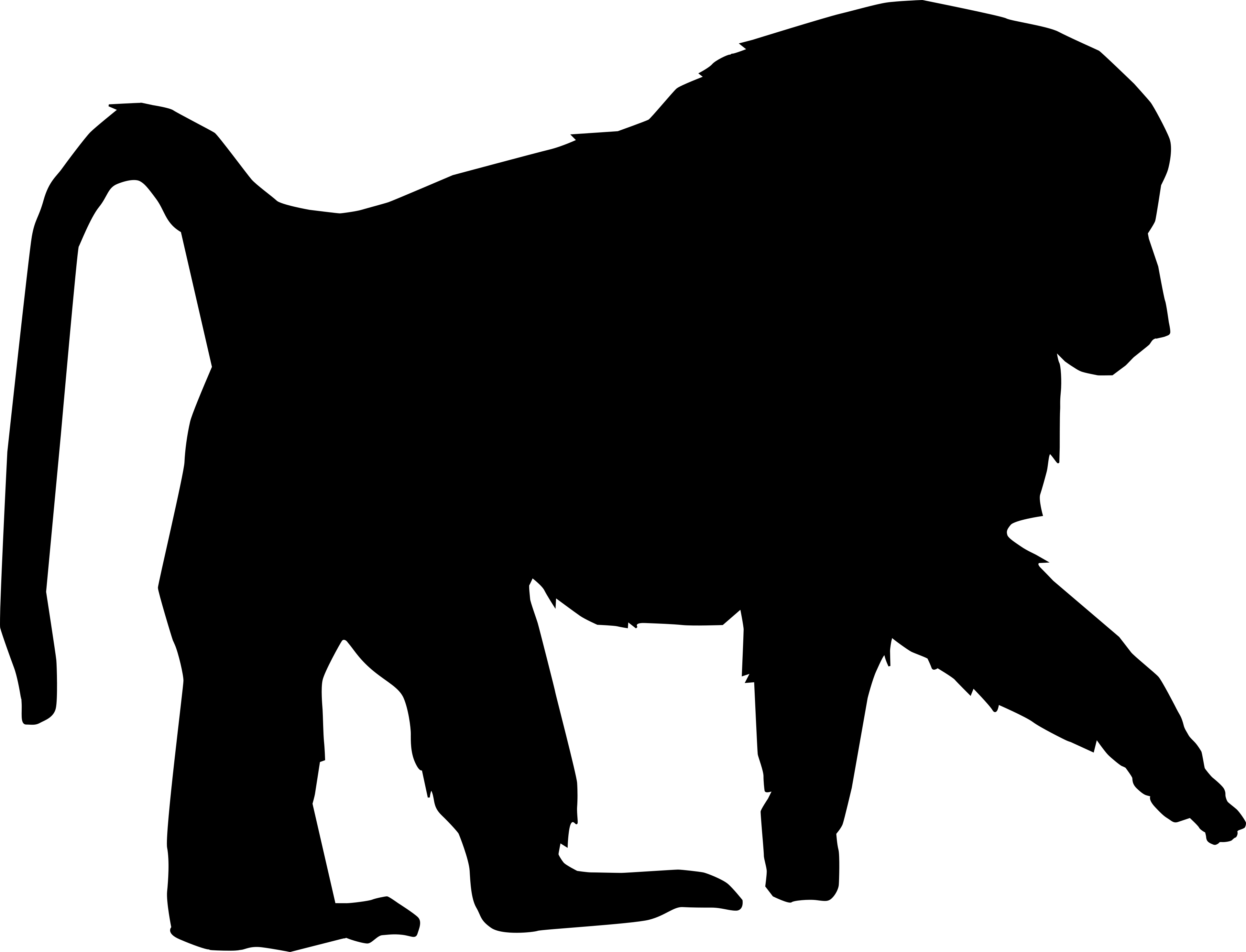

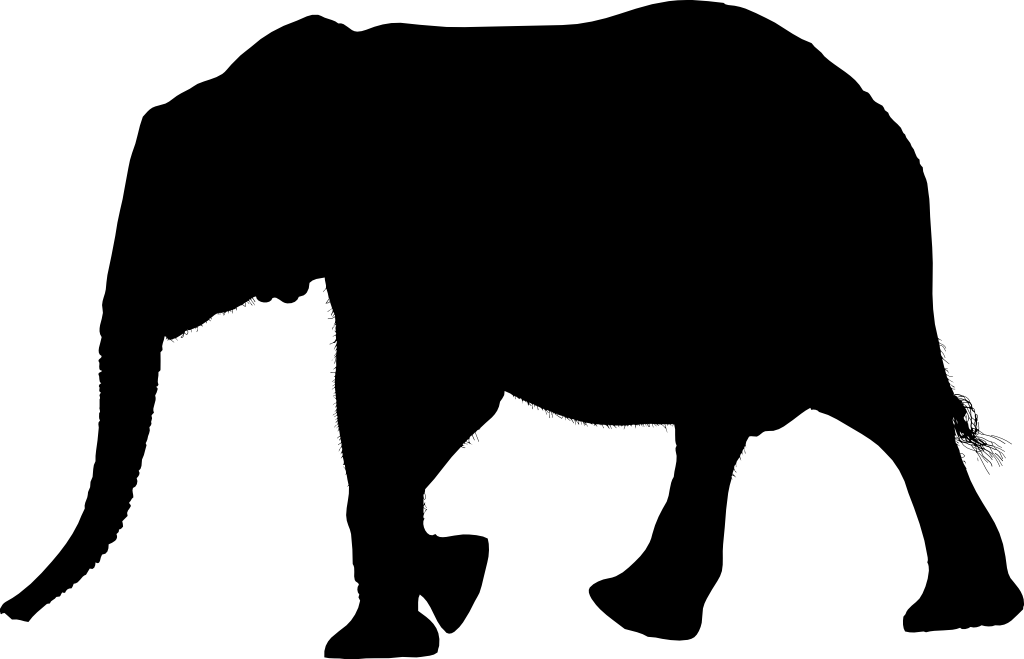

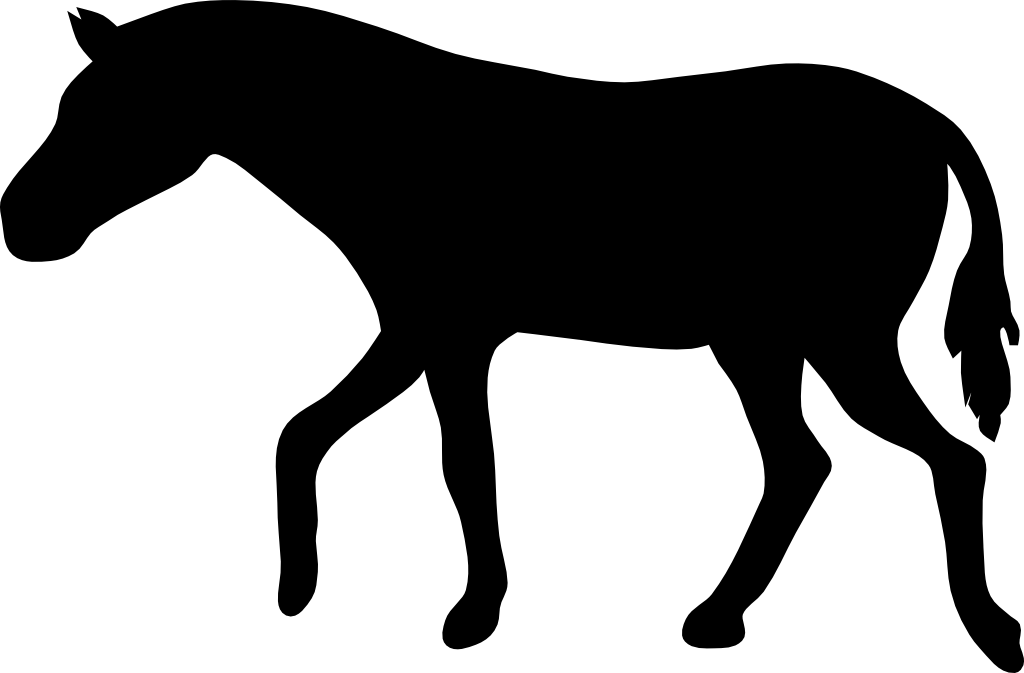

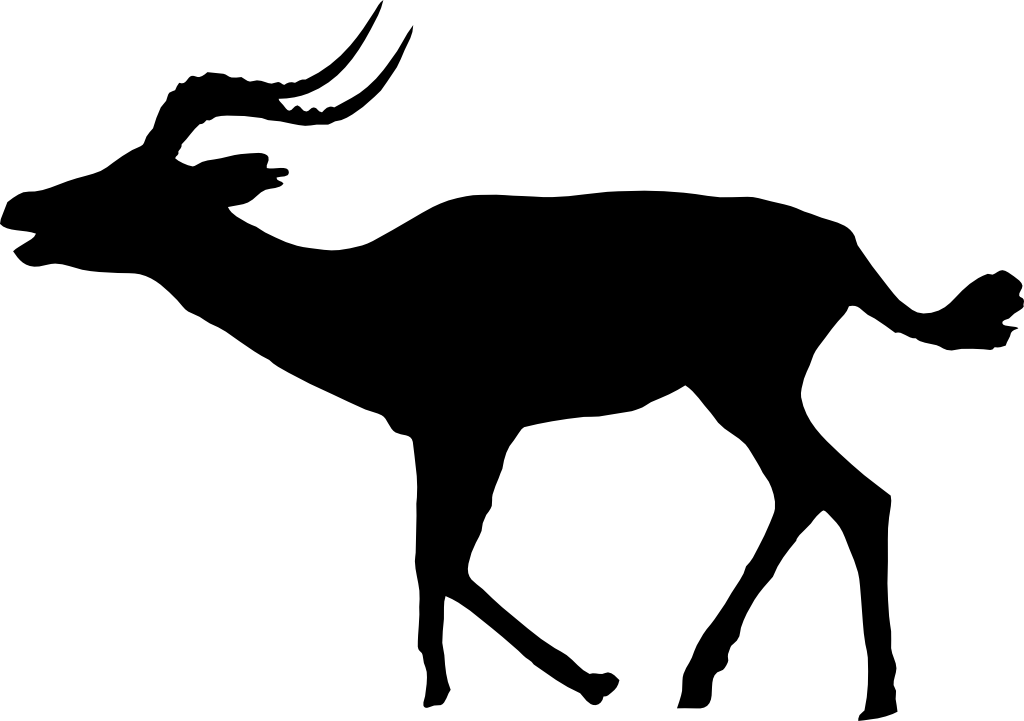

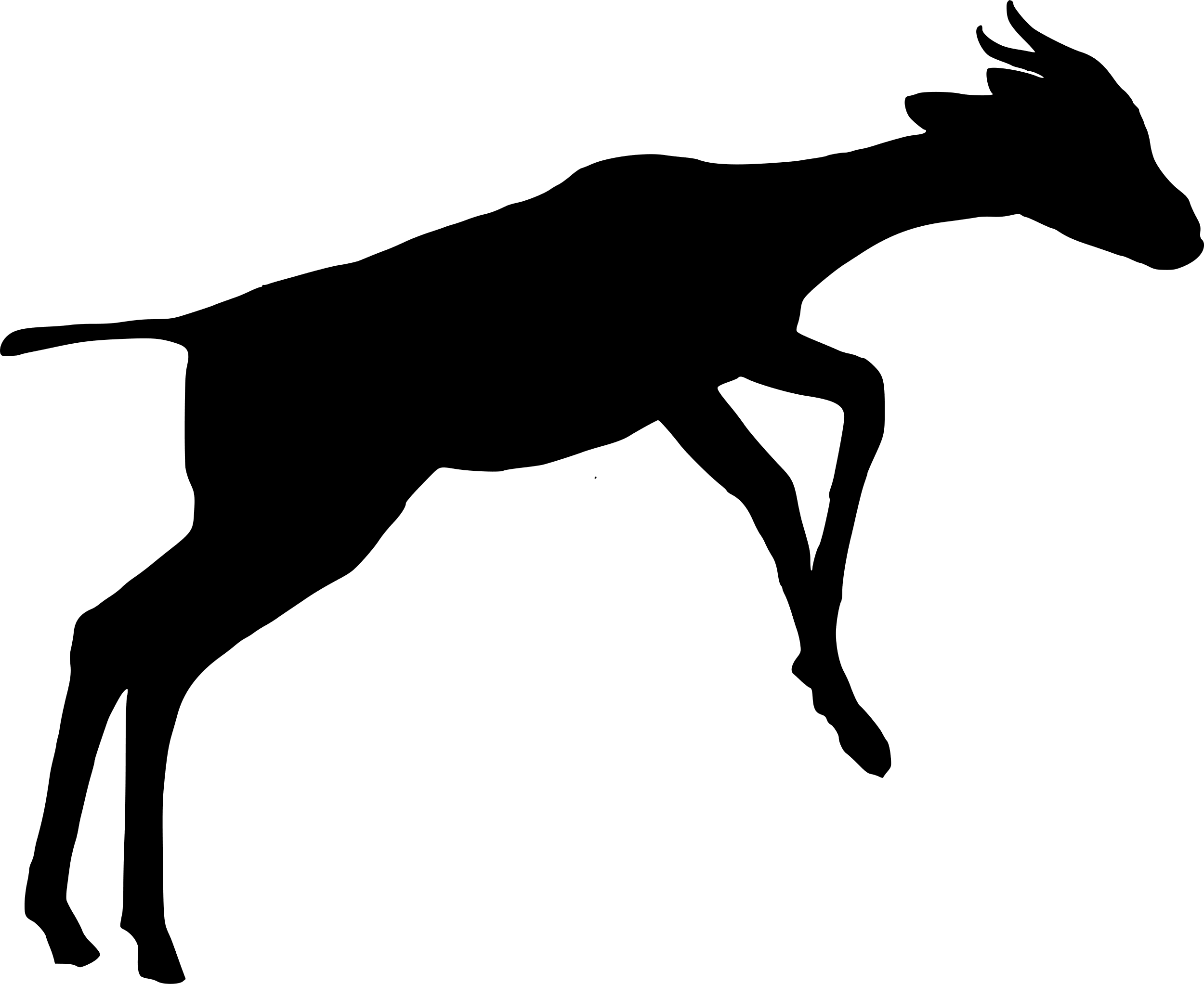

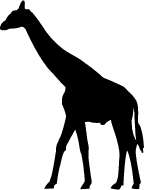

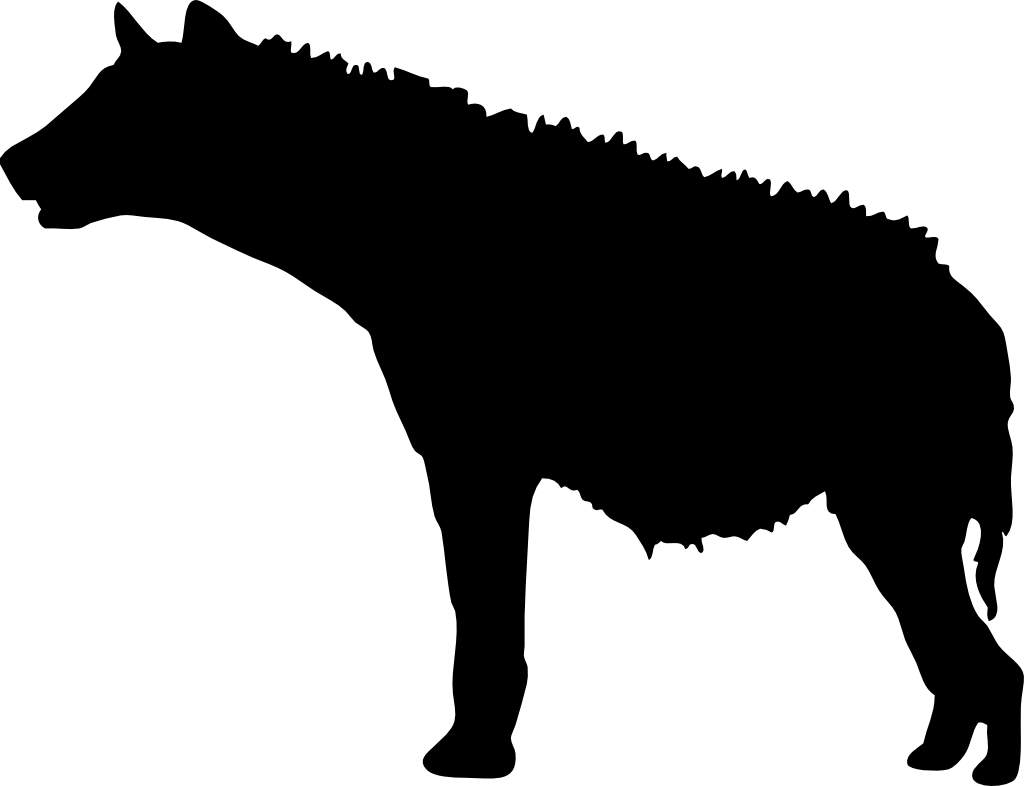

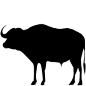

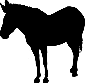

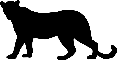


**Figure S7.** Marginal total effect of grid square-level *Opuntia* volume on the proportion of detections occurring at night for: **A)** olive baboon, **B)** vervet monkey, **C)** elephant, **D)** buffalo, **E)** dik-dik, **F)** impala, **G)** kudu, **H)** giraffe, **I)** Grevy’s zebra, **J)** plains zebra, **K)** spotted hyena, and **L)** leopard. The models assume that *Opuntia* indirectly affects occupancy through altering the composition of the native plant community; for model structure, see Figure 2 in main text. Shaded areas represent 89 compatibility intervals for the January-April (light green) and October-November (purple) seasons. Black lines indicate posterior median marginal effects for January-April under a new moon (―) and full moon (‧ ‧ ‧), and October-November under a new moon (– – –) and full moon (– ‧ –).
